# Adapting the Women’s empowerment in nutrition index: Lessons from Kenya

**DOI:** 10.1016/j.worlddev.2024.106887

**Published:** 2025-04

**Authors:** Erin Lentz, Nathan Jensen, Watson Lepariyo, Sudha Narayanan, Elizabeth Bageant

**Affiliations:** a Lyndon B Johnson School of Public Affairs, University of Texas at Austin, United States; b Global Academcy of Agriculture and Food Systems, University of Edinburgh, United Kingdom; c International Livestock Research Institute, Kenya; d International Food Policy Research Institute, India; e Independent Researcher, United States

## Abstract

Women face a disproportionate burden of malnutrition and food insecurity. Research has shown that women’s empowerment can buffer women against nutritional problems. This paper contributes to ongoing efforts to measure women’s empowerment that are both context-sensitive and universal, focusing on the recently developed Women’s Empowerment in Nutrition Index (WENI). Earlier research has shown it is both a valid construct and positively related to dietary and nutritional outcomes of women in South Asia. We establish that WENI is generalizable to agropastoral and pastoral Kenya, an area with substantially different livelihoods, food system, norms, and institutions than South Asia. We find that a locally contextualized WENI is strongly associated with women’s body mass index and dietary diversity as well as household level food insecurity. We also present findings for two shorter variations of WENI: an abbreviated WENI (A-WENI) and a cross context WENI (CC-WENI). A-WENI contains a small subset of WENI indicators identified using machine learning with South Asian data and therefore is context-specific. CC-WENI does not contain indicators specific to the validation context. We find that they perform comparably well with caveats. Thus, as use of WENI expands we recommend adapting WENI for in-depth analyses of women’s nutritional empowerment; using CC-WENI for cross-context comparisons; and using A-WENI for rapid appraisals of community level progress in a given context.

## Highlights

• Women’s empowerment has been shown elsewhere to be important to women’s own diet quality and nutritional outcomes.
• A tension for creators of empowerment measures is balancing context specific and generalizable indicators.
• We adapt the Women’s Empowerment in Nutrition Index (WENI) to northern Kenya, home to pastoral and agropastoral households.
• It is positively associated with women’s body mass index and dietary diversity, and with the reduced coping strategies index.
• We validate two alternatives, a cross-context WENI and an abbreviated WENI, and offer recommendations on their use.

## 1 Introduction

Hunger and undernutrition disproportionately affect women (UN Women, 2012, UNICEF, 2023). Recent research has identified women’s empowerment as a critical factor for the dietary and nutritional outcomes of women and their families (Herforth and Harris, 2014, Kadiyala et al., 2014, Malapit et al., 2015, Narayanan et al., 2019, Santoso et al., 2019, Quisumbing et al., 2021). However, empowerment is a complex, multidimensional concept, and there are numerous efforts to operationalize its measurement (see Pratley, 2016, Santoso et al., 2019 for reviews). One such measure is the Women’s Empowerment in Nutrition Index (WENI), created to understand the barriers and opportunities women face in achieving adequate nutrition (Narayanan et al. 2019). Developed and externally validated in rural South Asia, in contexts of widespread undernutrition, WENI has been shown to be strongly associated with body mass index, anemia and dietary diversity scores for women and men there (Narayanan et al., 2019, Narayanan et al., 2022). We apply WENI in East Africa for the first time, assessing it against anthropometric and dietary diversity outcomes.

WENI differs from other empowerment indices in several ways. First, it is comprised of nutrition-centered indicators resulting in an index. WENI’s choice of indicators is guided by the concept of nutritional empowerment, defined as the process by which individuals acquire the capacity to be well fed and healthy (Narayanan et al. 2022). This includes gaining access to, and control over, health and fertility, and intake of food which is adequate and nutritious; having knowledge about, and say over, health, fertility and nutritional practices; and being able to draw support from both family and other institutions in securing and maintaining an adequate diet and good health. It also conceptualizes empowerment to be not only women’s agency but also the resources and knowledge that help them to achieve their desired outcomes (Kabeer 1999).

Importantly, it has also been validated against (meaning correlated with) respondents’ *own* dietary and nutritional outcomes, which are intrinsically important (Narayanan et al. 2019). This is in contrast to other empowerment measures that often focus outcomes at either the household level or for other family members, such as children (Santoso et al. 2019).

WENI’s indicators’ focus on nutrition leads to a second important difference. WENI is applicable to all women, indeed to any individual. WENI does not require specific household compositions (e.g., some indices rely on responses to questions by spouses or partners, for example the survey-based women’s empowerment index (SWPER) (Ewerling et al., 2020; see also Yount et al., 2018) nor is it constrained to specific livelihoods (e.g., the Women’s Empowerment in Agriculture Index (WEAI) and the project-level WEAI focus on agricultural livelihoods (Alkire et al., 2013, Malapit et al., 2015, Malapit et al., 2019) and the Women’s Empowerment in Livestock Index (WELI) focuses on livestock-based livelihoods (Galiè et al. 2019)). Both are common for several other popular empowerment indices applied to the health and nutrition space, albeit with mixed evidence (Pratley, 2016, Santoso et al., 2019, Quisumbing et al., 2021). The recently developed Health and Nutrition Module, additional to the project-level Women’s Empowerment in Agriculture Index, measures women’s health and nutrition agency and is a notable exception although unlike WENI it is not an index and is focused on women’s agency rather than on a suite of empowerment aspects (Heckert et al. 2023).

In this paper, we seek to determine whether WENI is generalizable outside of South Asia, where it was developed and validated. To do so, we test WENI among agro-pastoralists and pastoralists in Samburu County, Kenya. The substantially different livelihood strategies and gender norms in Samburu provide an opportunity to assess the broader applicability of WENI in a location where undernutrition remains a problem. To apply WENI in this new setting, we used existing literature, key informant interviews, and focus group discussions to verify the relevance of the indicators that compose WENI. Through this process, six of WENI’s 45 indicators were adapted to reflect contextual differences. We then collected the revised set of WENI indicators from a sample of women in Samburu County and ran a series of regressions to understand the relationship between WENI and body mass index, diversity of diet, and household food security.

While testing the relationship between diet and nutrition and WENI is the primary objective of this study, we also tested the relationship between our outcomes and two shortened versions of WENI, both of which were constructed from a sub-set of questions in the WENI survey module. The first, which we call abbreviated WENI (A-WENI), was developed explicitly to reduce the survey burden on the participants. The second, which we call cross-context WENI (CC-WENI), was constructed from the sub-set of survey questions that are not context specific and offers an opportunity to generate a comparable empowerment index with less pre-survey field work. All three indices will be described in greater detail in the methods section.

In what follows, we first provide an overview of WENI and how it is constructed. We then describe our site in Section 3, and our data and methods in Section 4. In Section 5, we present our findings. We close with a discussion of our findings and their implications for those considering whether and how to apply WENI in new contexts.

## 2 Conceptual framework

WENI builds on a theoretically grounded and carefully constructed, conceptual framework that brings together empowerment theory (Kabeer 1999) and the UNICEF framework for achieving adequate nutrition (UNICEF 2018a). Many empowerment measures linked to health and nutrition, including WENI, draw on the seminal work of Naila Kabeer (1999) to conceptualize empowerment (cf. WEAI (Alkire et al. 2013); SWPER (Ewerling et al., 2020); project level WEAI (Malapit et al. 2019); WENI (Narayanan et al. 2019)). Kabeer argues that empowerment is the ability to exercise choice, which depends on a person’s resources, agency, and achievements. WENI is a multidimensional empowerment measure, capturing the dimensions of knowledge (a type of resource), resources, and agency. In WENI, these three dimensions of empowerment are applied to the UNICEF framework, which identifies institutions, food security, health, and fertility, as domains that are necessary for women’s nutrition.

**Fig. 1:**
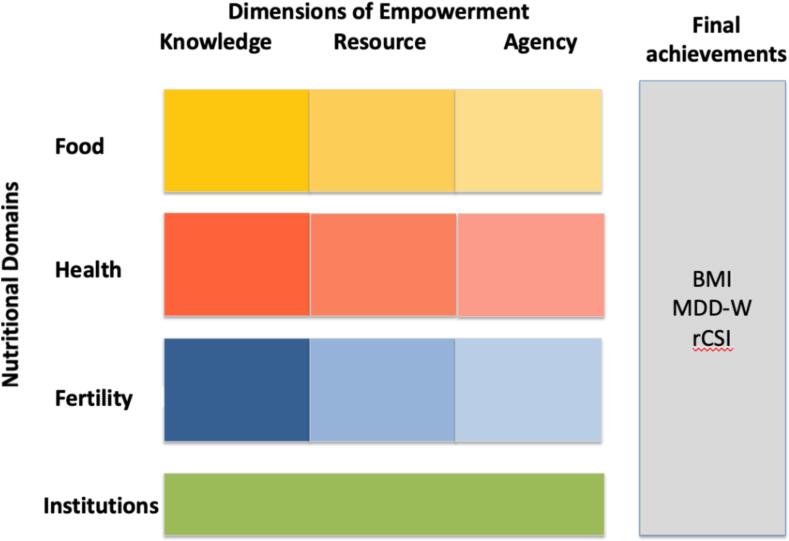
The ten domain-dimensions of WENI.

Fig. 1 shows the women’s empowerment in nutrition grid, which is composed of the three dimensions of empowerment interacted with four domains of nutrition yielding ten domain-dimensions (DD).^1^ The WENI DD provide a multidimensional structure for measuring empowerment, covering aspects of knowledge, resources, and agency cutting across basic, underlying and immediate causes of malnutrition. In WENI, institutions are considered to cut across the dimensions of empowerment, and for this reason is only one rather than three DD. Included in institutions are broad-based community norms (see Narayanan et al. 2022).

A total of 45 independent indicators populate the ten DD. Achievements are the nutritional outcomes of interest. Following Alkire and Foster’s (2011) multidimensional poverty measures, the index is additive and decomposable. Decompositions across domain-dimensions provide insights into which DD may be particularly low for a subpopulation or individual (see Lentz et al. 2021).

## 3 Site selection

**Fig. 2:**
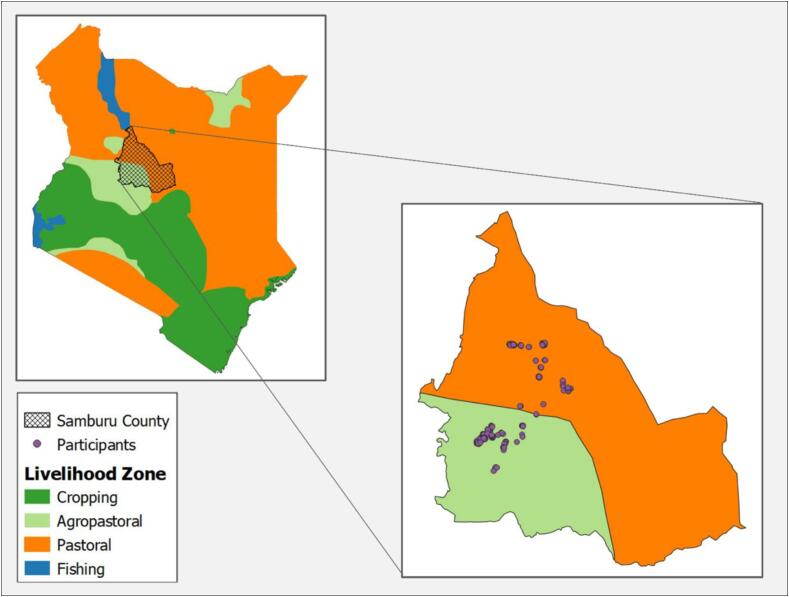
Locations of respondents in Samburu County, Kenya.

WENI was developed in rural South Asia, drawing on qualitative findings from rural Bangladesh and rural Odisha and Bihar in India. There is variation across these sites with respect to religion, availability of healthcare and other services, and state institutions; however, most rural residents were engaged in rain-fed agriculture, and many were vegetarian (Lentz et al. 2019). To assess whether WENI works outside of South Asia, we chose to apply WENI in a very different historical, cultural, and environmental setting, among pastoralists living in the arid and semi-arid lands (ASAL) in Africa, but one where undernutrition continues to be a formidable challenge. ASALs are 43 percent of Africa’s land area and home to more than 265 million pastoralists (African Union 2010). Samburu County is in ASALs of northern Kenya and is home to approximately 310,327 people (Kenya National Bureau of Statistics, 2019), most of whom engage in pastoralism or a combination of cropping and pastoralism (Fig. 2). It therefore provides an opportunity for assessing the index in an area where cropping is not the dominant livelihood and where households consume meat and meat-products. Further, Samburu County is remote, meaning that accessing services can be challenging for much of the population living there (Straight et al. 2016). For example, the first paved road linking the county capital of Maralal to a neighboring county was completed in 2022. In Kenya, ASAL residents are at greater risk for nutritional deficiencies than those living in non-ASAL regions. Women’s micronutrient status and dietary diversity in the ASAL areas are the worst in Kenya (UNICEF, 2018b, Kenya Demographic and Health Survey, 2015). Samburu has higher poverty and malnutrition rates than the Kenyan average (KDHS 2015).

The choice of Samburu County allows us to consider the intersection of gender and livelihood strategies. While gender on its own is an important pathway by which rural livelihoods can influence diet and nutrition (e.g., Kadiyala et al. 2014), livelihood strategies also influence nutrition. Fratkin et al. (1999) document how child nutrition varies based on livelihoods, foods produced and access to social services, all of which differ between rural, pastoralist households and sedentary households with some access to agriculture or with some access to day labor. In a sample from northern Kenya, they find that children in pastoral households are likelier to have higher protein intake but lower calorie diets compared to children in sedentary households and that children in sedentary households were likelier to experience faltering growth. We therefore also test for heterogeneity in the relationships between WENI and nutritional status across livelihoods.

Samburu shares some drivers of gender inequality with South Asia; other aspects differ (Kandiyoti 1988). Similarities include, for example, lower literacy rates for women than men, and, on average, a preference for boy children (Sen, 2005, Kock and Prost, 2017, Hoq, 2020). However, Walker (2021) has argued that pastoral women are doubly marginalized due to both their gender and their livelihood. Pastoral women have limited opportunities to own land and livestock, and in part because of low population density, often have less access to healthcare, education and employment opportunities than women in agricultural areas (Walker, 2021, Walker et al., 2022). Samburu County has a high fertility rate of 6.3, compared to an average of 3.8 in Kenya, 2.3 in India and 2.1 in Bangladesh in 2015 (World Bank. 2023, 2023, Kock and Prost, 2017). In Samburu, maternal mortality rate is 472 deaths per 100,000 live births, compared to Bangladesh’s rate of 200, India’s rate of 158, and Kenya’s rate of 353, all for 2015 (World Bank. 2023, 2023, Kock and Prost, 2017). The high maternal mortality rate reflects limited access to reproductive health care, with facilities sometimes a day’s walk away, among other factors (Macharia et al. 2022). High birth rates may reflect limited access to family planning, preferences for larger families, and low educational attainment (Straight et al., 2016, Kock and Prost, 2017, Lowe et al., 2021). Compared to South Asia, polygamy is more common in Samburu, but women have greater freedom of movement (Ramalingaswami et al. 1997). Lentz et al. (2019) found that many rural women in South Asia ate “last and least” meaning women often reported eating the leftovers after others had had their fill. In contrast, while food practices are related to age and gender in Samburu, Holtzman (2009) explained that men are expected to eat with restraint, “to safeguard the wellbeing of women and children” (p. 141). Thus, the different livelihood strategies available in Samburu relative to South Asia and variation in forms of gender inequality presents an important site for validating WENI.

## 4 Methods and data

To determine whether WENI is generalizable to a substantially different population than from rural South Asia, we adapted WENI to the new context. We estimated the association of our continuous and binary WENI scores with our achievements: body mass index and Minimum Dietary Diversity for Women (MDD-W). We also examined the association between the continuous and binary WENI scores and the reduced coping strategies index, a household measure of food insecurity (Maxwell and Caldwell 2008). We note that each outcome has limitations, discussed below.

We then examined associations between our achievements and two other WENI metrics: A-WENI and CC-WENI scores, both of which are subsets of the adapted WENI index to investigate whether either are suitable replacements for the longer WENI. Collecting empowerment information is costly both in terms of respondent time and resources (Bageant et al. 2024). Both versions of WENI allow us to test the degree to which a shortened version of WENI performs relative to the long form. A-WENI was developed using data from India and machine learning data reduction techniques (Saha and Narayanan 2022). Donald et al. (2020) has argued that there is a need for both locally relevant and cross-culturally comparable measures. In contrast to the adapted WENI, CC-WENI is a set of measures identical across contexts and excludes locally contextualized indicators.

The WENI survey module and anthropometric measurements were fielded in the third wave of an ongoing multiyear survey run by the International Livestock Research Institute (ILRI) to assess the impacts of a randomized control trial, entitled “Can Asset Transfer & Asset Protection Policies Alter Poverty Dynamics in Northern Kenya?” (Jensen 2019).^2^

***Indicators Assessed and Updated***

The original WENI index is composed of 45 binary indicators spread across ten domain dimensions. We expect the domain dimensions to be appropriate across contexts and generalizable. However, *a priori*, we did not expect the indicators within each domain-dimension to be universal for at least two reasons. First, the nature of gender inequality and patriarchy, which several of the indicators target, vary by location, although there are significant commonalities and overlap (Kandiyoti, 1988, Kabeer, 2016). Second, local institutions, knowledge, opportunities for agency, and resources vary across sites and between populations (e.g. by vulnerability status) within sites (Yount et al., 2018). Therefore, we assessed each indicator for appropriateness for the Samburu context and revised those that were not. The intention was to maintain the domain dimensions while allowing for flexibility in how that domain dimension was measured within the context.

Drawing on a literature review on gender roles and nutritional issues in ASAL areas and Samburu Kenya and interviews with local experts and researchers working on empowerment measurement, we reviewed indicators from the original WENI survey (McPeak and Doss, 2006, Holtzman, 2009, Lentz et al., 2016, Straight et al., 2016, Kock and Prost, 2017, Walker, 2021). We assessed each indicator using the following criteria: (1) easy to comprehend for Samburu respondents, (2) reliably reported by respondents as having a consistent meaning and likely to generate an accurate response, and (3) globally recognized as important for women (e.g., access to healthcare) or contextually appropriate (e.g., including access to MPesa, a cellphone based banking system active in Kenya).^3^ In this way, we revised indicators to clarify language and identified potentially sensitive indicators and their candidate replacements. Three of the authors then piloted original and revised indicators, findings from which resulted in a list our retained indicators (Collins 2003).

We replaced six indicators (see Annex Table A1 for original and replacement indicators). One question about knowledge of menstrual cycles and pregnancy was culturally sensitive, raising both ethical and data quality concerns. In Samburu, asking women if they are pregnant is perceived as bringing bad luck, particularly if the pregnancy is not visible, and few talk about menstruation with strangers. The remaining five indicators were not relevant (e.g., kwashiorkor is a more common nutritional problem in Kenya than calcium intake, which is a more common issue in South Asia) and had little to no variation in our pilots. Candidate replacement indicators were chosen because they fell within the same domain-dimension as the original indicators. We identified an Institutions domain question on social norms of hairstyles to replace a question on veiling through focus group discussions and one author’s local knowledge. Respondents explained that Samburu women are expected to keep their hair short to help highlight their beaded necklaces. Yet, some focus group women had long hair or extensions, suggesting their ability to push against prevailing norms, and asking reasons why women had certain hairstyles was thus a useful question on intrinsic motivation. We chose not to include a replacement question for a Food Agency question on dietary restrictions, which had no variation in Samburu and did not seem to be relevant to the context, since we had adequate coverage in that domain-dimension. We included two additional indicators. The first is related to livestock management and sales, since livestock is an integral Food Resource in pastoral and agropastoral livelihoods. We included a question on cellular networks in the Institutions domain, reflecting that coverage in northern Kenya is more limited than in South Asia. We revised 13 indicators to include contextually relevant choice sets or to clarify language to improve comprehension.

To cognitively test the final set of indicators for clarity of understanding, response variation, and appropriateness, in December 2021, the authors then held two focus group discussions (FGDs) with women similar to future respondents. The authors tested the revised survey for reliability by asking attendees to listen to survey questions and then explain the meaning of the questions back to the team in their own words.

In sum, we aimed to keep the ten domain-dimensions, the type of information captured in each domain dimension, and number of indicators consistent, while adjusting specific indicators to ensure both relevance and appropriateness (Table 1). In the robustness section, we estimate CC-WENI to determine the importance of the contextualized questions in the performance of the adapted WENI. See Annex 2 for the WENI survey module.

**Table 1:** Comparing Indicators in WENI, Abbreviated WENI (A-WENI) and Cross-context WENI (CC-WENI).

**Domain Dimension**	**WENI Kenya Indicator**	**Description in WENI Kenya**	**Compared to Original WENI**	**A-WENI**	**CC-WENI**
**FOOD**					
**Food Knowledge**					
1	FKkwashiorkor	Knows that a child's hair turning light brown is indicative of a nutritional issue of some kind	REPLACED	Yes	No
2	FKiodine	Knows iodized salt prevents goiter	REVISED	Yes	Yes
**Food Resources**					
1	FRpaidwork	Does market work as an employee, for wages or salary, in cash or kind	RETAINED	No	Yes
2	FRtravelforwork	Acceptable for respondent to work outside of village	REVISED	Yes	Yes
3	FRland	Owns land or has access to the land she needs	REVISED	No	Yes
4	FRservedlast	Is not served last at least sometimes	REVISED	Yes	Yes
5	FRincomesources	Has diverse sources of income (at least 3 of 5, from the following: paid work, own account family enterprise, agriculture, transfers, remittances)	RETAINED	No	Yes
6	FRselfemployment	Has own farm or non-farm employment	RETAINED	No	Yes
7	FRprograms	Has consistent access to a government or NGO program in the past year	REVISED	No	Yes
8	FRsupportent	Has received support for own enterprise or believes she could receive it	REVISED	No	Yes
9	FRdiversity	Has at least 3 food resources activities (e.g., farming, bee keeping, poultry, gardens, large animal pastoralism, small animal pastoralism, fishponds)	RETAINED	No	Yes
10	FRlivestockcontrol	Has ever owned livestock and has at least some control over how it is managed or when to sell it	ADDITIONAL	No	No
**Food Agency**					
1	FAdecisionpaidwork	Decision to undertake or not paid work	RETAINED	No	Yes
2	FAassetconsent	The earnings from livestock owned by the respondent have not been used without her consent. If respondent does not own livestock solely or jointly, coding as disempowered	REVISED	Yes	Yes
3	FAagrisay	At least some say in kitchen garden decisions and or cultivation (includes livestock, fishing, poultry and beekeeping)	RETAINED	Yes	Yes
4	FAdecisionent	At least some say in major or minor decisions in family business not owned by the respondent	RETAINED	Yes	Yes
5	FAcashcontrol	Has independent source of income (earned, transfers, or remittances) and has some control over how to use it	RETAINED	No	Yes
**HEALTH**					
**Health Knowledge**					
1	HKdiarrhea	Aware of any causes of diarrhea	REPLACED	Yes	No
2	HKors	Aware of what ORS is for and when to administer it	RETAINED	Yes	Yes
3	HKmalaria	Aware of how malaria is transmitted	RETAINED	Yes	Yes
**Health Resources**					
1	HRworkintensity	The majority of the work is moderate, light or sedentary	RETAINED	No	Yes
2	HRassistwhensick	Respondent receives help with household chores when sick	RETAINED	Yes	Yes
3	HRsanitation	Household has all three: protected water source, improved toilet, and vented cooking area	RETAINED	Yes	Yes
4	Hrworkhours	Does less than 8 h of work per day	RETAINED	No	Yes
5	HRworkrisk	No physical injury risk exists in work respondent does	RETAINED	No	Yes
**Health Agency**					
1	HAalonefortreatment	If unwell and chores are done, children are cared for, and there is money for healthcare, can seek healthcare (i.e., you do not need permission)	REVISED	Yes	Yes
2	HApermission	Can visit the health facility alone (unsupervised) if needed	REVISED	Yes	Yes
3	HAdecideownhealth	Can make decision on own health	RETAINED	Yes	Yes
**FERTILITY**					
**Fertility Knowledge**					
1	TKprograms	Has knowledge about programs that help expectant mothers	REPLACED	No	No
2	TKpregdiet	Has knowledge that pregnant women should eat more	REPLACED	No	No
**Fertility Resources**					
1	TRassistance	Received any assistance from gov't during last pregnancy, either in cash or in kind	RETAINED	No	Yes
2	TRworkload	Did not undertake heavy physical activity after 8th month of pregnancy	RETAINED	No	Yes
3	TRworkshare	Other HH members took over some or all of respondent's work	REVISED	No	Yes
4	TRqualafford	Has at least 1 ANC option that considers very good quality and affordable/free	RETAINED	No	Yes
5	TRpregless	Ate a normal amount or more in general during her last pregnancy. Or has not been pregnant	REPLACED	No	No
**Fertility Agency**					
1	TAdelivpref	Respondent decided place of delivery of last child	RETAINED	No	Yes
2	TAchildnumber	Has at least some say in total number of children	RETAINED	No	Yes
3	TAchildspacing	Has at least some say in spacing between children	RETAINED	No	Yes
**INSTITUTIONS**					
1	Igroup	Can become a member of any group out of his/her own accord or would join if such a group was available in her community	REVISED	Yes	Yes
2	Ihair	Can grow long hair or not because of intrinsic motivation	REPLACED	Yes	No
3	Igovinfo	Has a mobile phone and receives information on government schemes	RETAINED	Yes	Yes
4	Iviolence	Does not experience domestic violence or if experiences severe domestic violence, has support	REVISED	No	Yes
5	Imobility	Has freedom to visit bank /MPESA branch unaccompanied and does not need permission to visit parents	REVISED	Yes	Yes
6	Icivic	Civic engagement: Has participated in at least one activity in the last five years (petition, protest, public meetings, representation to government officials, voted in elections)	RETAINED	Yes	Yes
7	Inetwork	There is cell network where respondent lives or regularly goes	ADDITIONAL	No	No
**Total # of indicators**			45	20	37

Notes: “Revised” describes indicators that capture the intent of the original WENI indicator but are adjusted to the Kenyan context (e.g., expanded choice set or rewording for clarity).

“Replaced” describes indicators that are substantively differ from the original WENI indicator.

“Retained” describes indicators that are identical to the original WENI indicator.

We excluded visibly pregnant women from the empowerment survey. However, for the reasons described above, some pregnant women are included in our sample. This could impact the relationship between BMI and WENI. For example, a pregnant woman may have a higher-than-normal BMI, due to physiological changes that result in pregnancy weight gain. In a small qualitative follow up study in June 2022, we re-interviewed 30 women who had been surveyed during February or March and found four had had been pregnant at the time of the survey. Because we do not know the entire sample of women who were pregnant at the time of the survey, we do not exclude women we later learned were pregnant.

***Constructing the Index***

WENI scores are calculated by first assigning survey responses to the 45 binary indicators into their domain-dimension. Every domain-dimension has at least two indicators. For each indicator, a score of zero means not empowered and a score of one indicates empowered. Within each DD, if the sum of the indicators for an individual is below half of the total possible score, the DD’s score is also assigned a zero, meaning not empowered. The DDs are equally weighted. They are summed and converted to a continuous score ranging between zero and one. For example, a score of 0.5 indicates that a woman is empowered in at least half of the domain dimensions. Applying a cutoff of 0.5 means if respondents’ scores are below 0.5, they are not empowered. For the binary WENI, respondents with scores greater than or equal to 0.5 are assigned a score of one, indicating “empowered” and those with scores below 0.5 are assigned a score of zero, indicating “not empowered.” A binary version of WENI can support evaluation.

We also assess two alternative versions of WENI: abbreviated WENI (A-WENI) and cross-context WENI (CC WENI). The two measures each help us understand different policy questions. First, collecting empowerment information is costly both in terms of respondent time and resources. The average time across respondents to complete the full WENI survey was 21 min, not counting the preamble and oral consent, (Bageant et al. 2024). This is lengthy, especially when surveys also include other questions. A-WENI allows us to test the degree to which a shortened version of WENI performs relative to the long form. Like WENI, A-WENI has ten domain dimensions and is a validated metric. The benefit of A-WENI is that it has fewer indicators in each DD with a total of 20 indicators (rather than 45), making faster to collect. In South Asia, it was found to be a valid measure of nutritional empowerment but performs differently from WENI when disaggregated for the study of domain-dimension specific outcomes (Saha and Narayanan 2022). Before applying it to the Samburu case, three indicators in A-WENI were replaced to reflect the Samburu context (the included indicators are FKkwashiorkor; HKdiarrhea; and Ihair). These changes are in Annex Table A1.

The second is cross-context WENI. While WENI includes some locally contextualized indicators, CC-WENI aims to provide a set of measures broadly applicable across contexts. The process of assessing indicators and contextualizing their related questions requires considerable time and resources, begging the question, what is the value contributed by the small subset of indicators that required substantial resources to adapt WENI to a local context? To answer this question, we constructed CC-WENI from the subset of indicators that required no contextualization—that were expected to be relevant and appropriate in both Samburu County and South Asia. In total, we excluded eight indicators that were replaced or additional (Table 1) to the original WENI. Importantly, CC-WENI excluded the only two questions from the fertility knowledge domain dimension. Both were adapted for the Kenyan context (i.e., on programs for expectant mothers (Tkprograms) and knowledge on dietary quantity (Tkpregdiet), reflecting that the original questions covered topics challenging to ask in the Samburu context. A result of excluding these two questions is CC-WENI has only nine DD. CC-WENI excluded the four replacement indicators, tailored to the Samburu context (FKkwashiorkor; Hkdiarrhea; Trpregless; Ihair) as well as the two new, additional indicators (FRlivestockcontrol and Inetwork). We found that some indicators in CC-WENI, for example the ability to seek healthcare without permission (i.e., HApermission), have limited variation in the Samburu sample but are important barriers to women’s agency in South Asia (Lentz et al. 2019). To enable future cross-context comparisons and to highlight factors that might be universally important (such as accessing healthcare without permission), we include them in CC-WENI although they are not highly variable in this context.

We include in our analyses below all four metrics: continuous WENI, binary WENI, and A-WENI, and CC-WENI. To understand whether these metrics differ, we estimate the same set of outcomes sequentially using each metric, and compare the results between the continuous WENI, A-WENI, and CC-WENI.

***Sample***

The adapted WENI survey module was collected February – March 2022 as part of an ongoing survey led by ILRI (Jensen 2019). The multi-year sample included 1,875 women from agro-pastoral and pastoral communities, including recently sedentarized households. One primary objective of the larger survey was to study the impacts of the Rural Entrepreneur Access Project (REAP), a program that uses entrepreneurial training and business grants in an effort to lift targeted women out of poverty by developing an additional livelihood activity. The larger survey sample was composed of two groups. 80 % of the sample were women that were eligible for the REAP program, which meant, among other things, that they had been identified by their community as falling within the lowest two wealth categories from a set of wealth categories developed by the community. The remaining 20 % were from the 3rd lowest wealth category, placing them just above the eligibility threshold for REAP. The ILRI study included core demographic information, livestock holdings, livelihood activities and consumption modules.

From the larger ILRI sample, this study sampled a subset of participants from three wards within Samburu: Ndoto, Elbarta, and Angata Nanyekie, the latter is agro-pastoral. Target sample sizes for each ward were set to ensure that about 40 % of the sample would be from an agro-pastoral region and 60 % from a more purely pastoral region. The process for selecting women to participate in the empowerment survey, which was collected as a separate survey from the larger multi-year survey, was as follows. At the end of the multi-year survey, the enumerators described the objectives of the empowerment study and explained that all women who were between 18 and 49 years old and who were not pregnant, were eligible to participate. Then the enumerator asked the respondent if she was eligible.^4^ To avoid survey fatigue among those eligible, enumerators scheduled a follow up time (usually the following day) to collect the empowerment survey. Our primary objective was to validate WENI in a population that was very different from that used to first verify the relationship between WENI and BMI, but also where undernutrition was pervasive. The pastoral population in Samburu was that population. We determined the target sample size of pastoralists by first doing a power calculation to identify the minimum sample needed to statistically identify a relationship between WENI and BMI, if the true relationship was the same as that found in India where that relationship was first verified Narayanan et al. (2019). We then doubled that sample so that we would be powered to separately test for the same relationship among agro-pastoralists in Samburu. The resulting minimum sample was 297. To allow for any errors or mis-surveys, we collected 338 surveys. In the analysis, we dropped eight women who were missing data on one or more factors used in the analysis and dropped three duplicate surveys. The final sample used in the analysis was 327. Of these, 119 had engaged in cropping at some point over the previous five years. Enumerators collected survey data by tablet. Descriptive statistics are presented in Table 2.

**Table 2:** Descriptive statistics: continuous variables report mean and standard deviation. Binary variables report counts and percentages.

**Variables**	**Pastoral** **(n = 205)**		**Agro-pastoral (n = 104)**		**Total** **(n = 309)**	
**Outcome**						
Mean Body Mass Index (BMI)	18.21	(2.35)	19.03	(2.25)	18.48	(2.35)
BMI Category (%)						
Severe thinness	15.61		6.73		12.62	
Moderate thinness	19.51		13.46		17.48	
Mild thinness	26.83		25.00		26.21	
Normal range	38.05		54.81		43.69	
Mean MDD-W^1^	2.05	(0.96)	2.17	(0.89)	2.09	(0.94)
Mean rCSI^2^	15.29	(10.29)	12.66	(10.75)	14.40	(10.50)
**Women Empowerment in Nutrition index (WENI)**						
Mean WENI	0.52	(0.11)	0.48	(0.13)	0.51	(0.12)
Mean CC-WENI	0.52	(0.11)	0.47	(0.13)	0.50	(0.12)
Mean A-WENI	0.49	(0.12)	0.46	(0.13)	0.48	(0.12)
WENI status (% empowered)	58.54		39.42		52.10	
**Other variables**						
REAP participant (% enrolled)	36.10		30.77		34.30	
Marital status (%)						
Never married	5.85		0.00		3.88	
Married	80.00		95.19		85.11	
Widowed	10.24		1.92		7.44	
Divorced	3.90		2.88		3.56	
Mean Age (years)	29.78	(6.10)	30.07	(7.03)	29.88	(6.42)

Note: Standard deviations are in parenthesis.

1 Minimum Dietary Diversity score- Women (MDD-W).

2 Reduced Coping Strategy Index (rCSI).

***Outcome Measures***

In addition to the WENI module, we also collected data used to compute our outcome measures: body mass index (BMI), Minimum Dietary Diversity for Women (MDD-W) and the reduced coping strategies index (rCSI) (see Table 2). While a relationship between empowerment and diet and nutrition has been widely documented, as Quisumbing et al. (2021) has argued, the relationship between empowerment and women’s own nutrition is complex. Different dimensions of empowerment can involve tradeoffs and the resulting explained variation in diet and nutrition outcomes associated with empowerment is often quite small (Quisumbing et al. 2021). While WENI was developed with a focus on nutritional empowerment, primarily to address this challenge, the fact remains that dietary and nutrition outcomes are influenced by several factors, many of which are difficult to observe or measure (Blackburn and Jacobs, 2014, UNICEF, 2018a). Thus, to provide breadth in our analysis and to acknowledge the different limitations of these outcomes, we present results on BMI, dietary diversity, and rCSI.

BMI has been used to set thresholds for underweight, overweight, and obesity in adults. BMI faces several limitations (Blackburn and Jacobs 2014). Nutritional outcomes – including body mass – are influenced by not only empowerment and diet but also underlying diseases, sanitation, and other factors (UNICEF 2018a). Further, small changes in BMI among underweight populations may not translate into significant improvements in health.^5^ Finally, BMI is a poor measure of health status for overweight and obese adults (Blackburn and Jacobs 2014). Nonetheless, BMI is commonly used tool in nutritional screenings to identify individuals who underweight, a serious health and nutrition concern in our sample (WHO 2023; UNICEF 2018b). To avoid interobserver variability, two trained members of each team collected weight and height measurements.

MDD-W is a continuous dietary diversity measure that counts food groups consumed by the surveyed women in the past seven days (FAO 2013). MDD-W has nine food groups and values can range from a low of zero to a high of nine and are thought to capture diversity of diet (Maxwell et al. 2014). MDD-W has been used in a range of other studies of other empowerment measures and in sub-Saharan Africa (e.g., Kassie et al., 2020, Onah et al., 2021) and beyond (e.g., Malapit et al., 2015, Sinharoy et al., 2018). It is valued as a low-cost proxy measure of diet quality and is on the pathway to nutritional outcomes but is less precise than comprehensive efforts to measure diet quality (FAO 2013).

Unlike BMI and MDD-W, which focus on respondents’ outcomes, the rCSI is a household level measure. The rCSI measures food insecurity by asking about the frequency of five food-related coping strategies (e.g., begging for food or reducing meals) undertaken in the last seven days (Maxwell et al. 2014). Values range between zero and 56, with higher scores indicating worse food insecurity. A score of 11 indicates food insecurity; a score of 18 indicates a high degree of food insecurity (Maxwell et al., 2014, IPC, 2021).

***Empirical Model***

Prior research has shown WENI is a valid construct and that the WENI index is related to dietary and nutritional outcomes. WENI was developed in South Asia with a focus on the relationship between nutritional empowerment and better dietary diversity, normal hemoglobin levels, and normal weight (i.e., not being underweight or obese). Anemia is not a significant issue in Samburu, but a large percentage of our sample was underweight; we therefore focus on whether WENI is associated with being not underweight and with having adequate dietary diversity, adding a household food security measure as an additional outcome indicator.

In this study, we test if the statistical relationship holds for these outcomes in a new context. We estimate our achievements as a function of WENI and a set of controls. WENI was designed to be related to nutritionally important measures for women themselves, and estimating these relationships provide insights into the predictive validity of WENI.^6^

(1) $$y_{w,i}=\alpha +\;\beta {\mathrm{WENI}}_{w,i}+\delta x_{w,i}+\theta_{w}\;+\varepsilon_{w,i}$$

We estimate three outcomes, indicated by $y_{w,i}$, where w indicates ward and i indicates the individual*.* The first outcome is a continuous measure of BMI. The second outcome is a binary BMI indicator where one is assigned to BMI values above equal to or greater than 18.5, which is the threshold for underweight (18.5) and zero otherwise. The third outcome is women’s minimum dietary score (MDD-W) from 24-hour consumption recall questions included in the survey.^7^ We also estimate a fourth outcome, rCSI, a household level measure of food security. In this case, *i* indicates the household. In all cases, β is the coefficient of interest.

The continuous WENI index for each household in each ward is ${\mathrm{WENI}}_{w,i}$. We include a series of controls, $x_{w,i}$, including age, age-squared, marital status,^8^ whether they were a participant in the REAP program, and an indicator if the household has grown crops within the five years leading up to the survey.^9^ All sampled households reported engaging in livestock-based livelihoods in the past five years, although a few had destocked/lost livestock at the time of the survey due to an ongoing drought. We include ward fixed effects, $\theta_{w}$*,* to account for prospective differences in BMI related to variation in environmental or institutional features across the landscape. As a robustness check, we include fixed effects at the manyatta level, which are similar to community or village level controls. Following Narayanan et al. (2019), we omit wealth levels from the regression specification because these differences in access to resources on account of differences in wealth are expressed adequately in the set of indicators of WENI, representing Food and Health resources.

We estimate linear regressions for the continuous measure of BMI and for rCSI, which is bounded by zero and 56 but whose distribution is close to normal within those bounds; a probit regression for the binomial measure of BMI; and a Poisson regression for the MDD-W measure, which is count data bounded by zero and nine. To reflect that the first stage of sampling for the empowerment survey occurred at the manyatta level, we cluster our standard errors at manyatta level for all models.

In the results that follow we exclude from our analysis four obese respondents (BMI >= 30) and 14 overweight individuals (BMI >= 25), dropping our sample to 309. In the robustness section, we present results from analysis that include them; results are similar. In general, WENI was constructed for rural undernourished populations and as such the link between WENI and obesity remains to be explored.

## 5 Findings

***Descriptive Data***

The prevalence of respondents with a BMI below the World Health Organization’s (WHO) cutoff for underweight (18.5) is 56 percent. This rate is consistent with the World Health Organization’s category of a critical public health situation (WHO 2023). Further, relative to the global population, our sample has high prevalence of BMI below 16, which is linked to increased risks for ill health and other adverse outcomes (World Health Organization, 2023). A multi-season drought that had impacted the nutritional outcomes of women and their families (FEWSNET 2022) was ongoing during the survey period. As a result, BMI is likely lower on average than would be during non-crisis periods. 61 percent of pastoral women are underweight; in contrast, 45 percent of agro-pastoral women are underweight. The MDD-W, which can range between zero and nine, is similarly low between the two livelihoods, both averaging just over 2 food groups. Mean rCSI scores are well above the threshold of 11, which indicates a high prevalence of food insecurity in the sample (IPC 2021).

The descriptive statistics table shows the mean WENI score is (coincidentally) 0.51, indicating that women, on average, are at the threshold for nutritionally empowered (set at 0.5). Separating by livelihood, the mean score for those that have recently grown crops is lower than those that have been purely pastoral (t-statistics = 2.67). The means and standard deviations of the CC-WENI and A-WENI are nearly identical to those of WENI. About 50 % of women in our sample are empowered in either of these three metrics, but this rate is higher among agro-pastoral women than among pastoral women. Ward level summary statistics, which show relatively low variation between wards, are presented in Annex Table A2. Summary statistics for each WENI indicator, including by pastoral and agro-pastoral subsamples are reported in Annex Table A3.

**Fig. 3:**
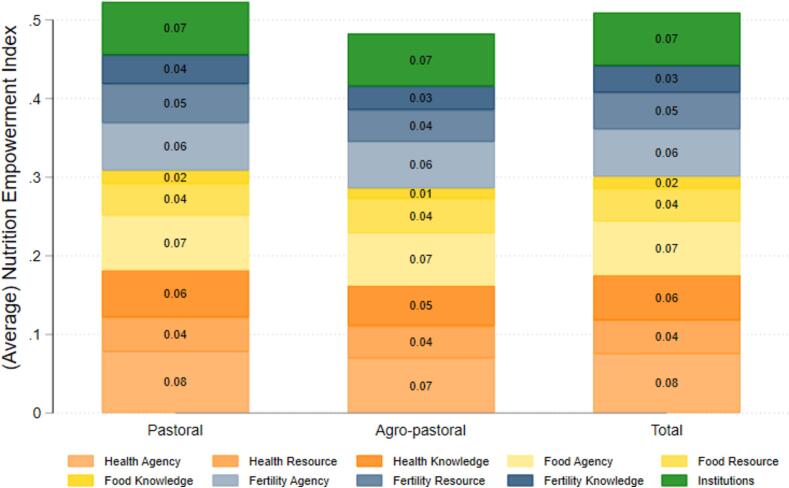
Decomposition of WENI scores into the ten domain-dimensions. Mean contribution of each DD to the total WENI score.

As described above, WENI is composed of ten domain dimensions. Fig. 3 presents a decomposition of the average scores across each of the ten DDs by livelihood status. The y-axis is the simple summation of these DDs. Food knowledge is the weakest domain in aggregate and in each livelihood. Institutions and health agency are the strongest.

Fig. 3 shows that women residing in pastoral areas have slightly higher domain dimension scores in several areas including health agency, health knowledge, food knowledge, and fertility resources than women residing in agro-pastoral areas. To test if these group differences are statistically significant, we use Wilcoxon ranked sum tests (Table 3). We find women residing in pastoral areas have higher levels of health agency (p-value = 0.00), health knowledge (p-value = 0.03), and fertility resources (p-value = 0.00) than women residing in agro-pastoral areas at a five percent level of statistical significance. In all domain-dimensions, the two groups are not significantly different. A reason why women residing in pastoral areas may have more health agency and knowledge is that their partners are often away from home in satellite camps with their animals for many days, leaving women in charge of day-to-day decisions regarding health seeking. In contrast, women residing in agro-pastoral areas are more likely to have a spouse at home most of the time, which may decrease their opportunities for agentic action. This merits further investigation.

**Table 3:** Wilcoxon-ranked-sum (Mann-Whitney) test values of pastoral women compared to agro-pastoral women.

**Domain-Dimensions**	**Pastoral**	**Agro-pastoral**	**Z-statistic of ranked sum test**	**P-value of ranked sum test**
Food	0.42	0.41	0.32	0.75
Food Agency	0.70	0.67	1.40	0.16
Food Resources	0.41	0.44	−1.58	0.11
Food Knowledge	0.16	0.13	0.95	0.34

Health	0.61	0.54	3.41	0.00
Health Knowledge	0.60	0.52	2.16	0.03
Health Resources	0.43	0.41	1.42	0.15
Health Agency	0.78	0.70	3.20	0.00

Fertility	0.49	0.43	2.62	0.01
Fertility Agency	0.60	0.59	0.37	0.71
Fertility Resources	0.50	0.40	3.52	0.00
Fertility Knowledge	0.37	0.30	1.88	0.06
				
Institutions	0.67	0.67	0.06	0.95
N	205	104		

***Core model results***

We now turn to our main results. We estimate BMI, a binary BMI variable (where 0 = underweight), MDD-W and rCSI, using our continuous WENI variable. The abridged results of the analysis are provided in Table 4. The full results are provided in Annex Table A4.

**Table 4:** Regression estimates from Equation 1 for outcomes BMI, binomial BMI, and MDD-W and rCSI with WENI as a continuous variable. Coefficient estimates are presented for OLS models and average marginal effects are presented for Probit and Poisson models.

**Variables**	**BMI (OLS)**		**Normal BMI = 1 Probit)**		**MDD-W (Poisson)**		**rCSI (OLS)**	
WENI	2.679**	2.671**	0.775	0.712	0.763***	0.863***	−10.14*	−13.70**
	(1.027)	(1.039)	(0.607)	(0.621)	(0.238)	(0.237)	(5.621)	(5.452)
Observations	309	309	309	309	309	309	309	309
Ward FE	YES	YES	YES	YES	YES	YES	YES	YES
Controls	NO	YES	NO	YES	NO	YES	NO	YES
R^2^-[Pseudo-R]	0.024	0.072	[0.010]	[0.039]	[0.005]	[0.010]	0.047	0.091

Notes: *** p < 0.01, ** p < 0.05, * p < 0.1.

Controls include age, age squared, marital status, an indicator on cropping for the 5 years of survey rounds, and participation in the REAP program.

Standard errors in parentheses are clustered at the manyatta level.

The results in Table 4 show highly statistically significant relationships between WENI and the continuous measure of BMI, MDD-W and rCSI. Both BMI and MDD-W are positively associated with increases in WENI. The rCSI is a household measure of food insecurity. We find that increases in WENI are associated with decreases in food insecurity, a relationship in the expected direction. Including control variables does not substantially impact the coefficient estimates’ size or their precision. When BMI is converted to a bivariate variable, where being underweight (BMI < 18.5) is given a score of zero and normal BMI is scored 1, the coefficient on WENI in no longer statistically significant potentially reflecting on the density of the BMI distribution relative to the cut-offs (Annex Figure A1). This is consistent with findings in South Asia, where women’s continuous WENI scores were strongly associated with BMI but not with BMI converted to a bivariate variable (see Nayaranan et al. 2019). Outcome indicators that employ cutoffs lose the precision captured by continuous measures.

**Fig. 4:**
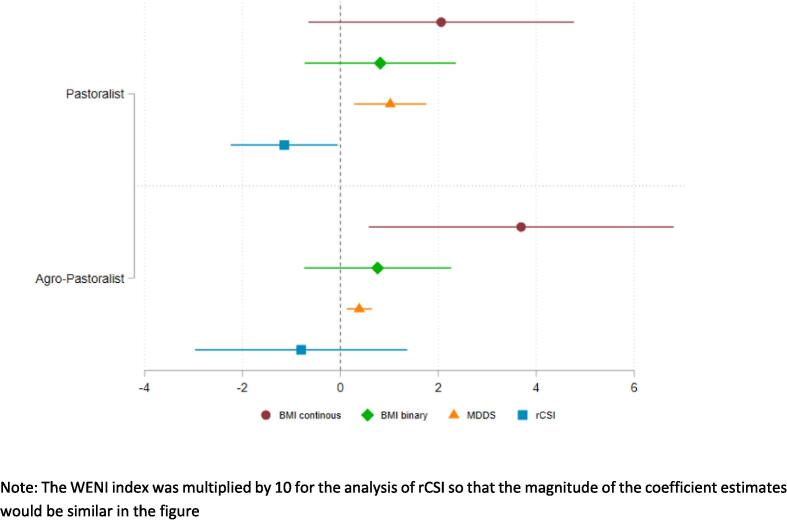
**Testing for heterogeneity in the relationship between WENI and nutritional outcomes by livelihood group.** Note: The WENI index was multiplied by 10 for the analysis of rCSI so that the magnitude of the coefficient estimates would be similar in the figure.

Earlier research found that child nutrition varied across livelihoods in northern Kenya (Fratkin et al. 1999). Further, WENI was developed in areas of South Asia where cropping agriculture was the primary livelihood strategy. To test if the relationship between WENI and nutritional outcomes are mediated by livelihood, we re-estimate equation 1 using the same outcomes as Table 4, but allowing for heterogeneity in β between those that have and those that have not reported growing crops. Figure 4 presents the abridged results of that analysis, showing the estimates for each group separately and testing each of them against the null hypothesis that they are zero, at the 5 % level of statistical significance. We caution that our study is not well powered for this test unless the differences are extremely large.

The estimates illustrated in Fig. 4 and presented in Annex Table A5, indicate that the relationship between WENI and the outcomes are qualitatively similar by livelihood type —WENI is associated with higher BMI, higher MDD-W and lower rCSI—although there is some heterogeneity in the magnitude and consistency of those relationships. Understanding heterogeneity by livelihood strategies remains an important area for further research.

***Headcount WENI and thresholds***

Binary indicators, such as headcount poverty, are often helpful for targeting interventions and to track the progress of the community. However, the cutoffs of indices are often researcher-determined, raising a question of whether the results are highly sensitive to the threshold chosen (Alkire et al., 2013, Malapit et al., 2019, Narayanan et al., 2019). To understand how sensitive the relationships between the binary WENI-empowered indicator and outcomes are to the choice of threshold, we create a series of binary measures of empowerment, where respondents with a WENI score of greater than a certain value (e.g., 0.5) are assigned an empowerment score of one and those below the value are assigned an empowerment score of zero. We then vary the threshold and examine the estimated relationships between empowerment and the outcomes for sensitivity to the threshold. In this case, the threshold is varied from 0.3 to 0.7. The estimates are generated using the model described by equation 1, substituting the binary WENI-empowerment indicator in place of the continuous ${\mathrm{WENI}}_{w,i}.$

**Fig. 5:**
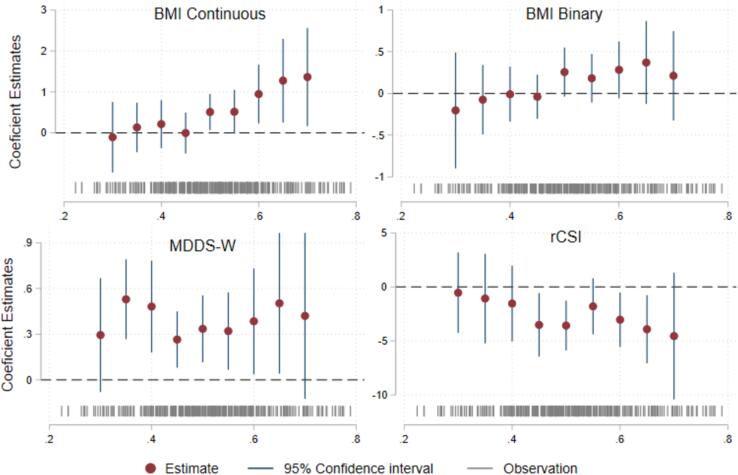
The coefficient estimates and 95% confidence intervals of outcome variables.

Fig. 5 illustrates the sensitivity of the coefficient estimates relating a binary indicator of empowerment to outcomes, to the threshold in WENI used to distinguish between those that are empowered and those that are not. First, focusing on the results related to the suggested empowerment threshold of 0.5, we see that the coefficient estimates broadly agree with those generated using the continuous index (Table 4). Second, the relationships between the binary WENI indicator of empowerment and rCSI and MDD-W change very little when the threshold is shifted within reason. Neither coefficient estimates change signs when the threshold is adjusted +/- 10 % around the suggested threshold of 0.5 (i.e., ranging from 0.40 to 0.60). This shift changes the ratio of women designated as empowered from 83 % to 19 %, changing the status of 64 % of the sample. Changing WENI thresholds does change the sign on continuous BMI and the relationship is stronger as thresholds are increased. However, as shown in Table 4, we caution that the coefficient on WENI is not statistically significantly associated with binary BMI. Third, the indicators of empowerment have the most power near the suggested threshold of 0.5. The general loss of precision in the estimates as the threshold moves away from 0.5 could be driven by variation across that index in the relationships between the WENI index and outcomes, or from a general loss of power as the number of observations within a category (empowered or unempowered depending on which threshold is being used) becomes small.

Figure 5 shows signs on the coefficients are not highly sensitive to the thresholds selected (see also Bageant et al. 2024). Thresholds are often chosen as a matter of convenience or ease of interpretation. Meaningful thresholds likely vary from one place to another and should be aspirational (Alkire et al. 2013). In our sample, which is relatively small, we see relatively few respondents at either end of the range. See Annex Figure A1 for kernel density of BMI by empowerment status. We therefore do not put strong emphasis on changes in the estimates because they are nearly statistically indistinguishable from one another.

***Reduced forms: A-WENI, CC-WENI***

**Fig. 6:**
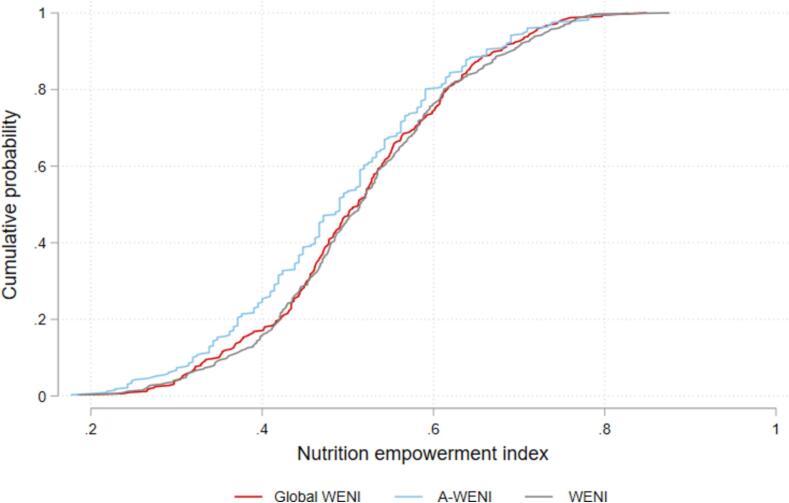
Cumulative distribution functions of WENI, A-WENI and CC-WENI.

We now turn to assessing the two shortened forms of WENI—A-WENI and CC-WENI. Both have the potential to save researchers considerable resources and reducing the burden on survey respondents. Fig. 6 illustrates the cumulative distribution functions of the three indices. Visually, A-WENI appears to consistently rank respondents as less empowered than the other two indices. Kolmogorov – Smirnov tests for equality between distributions found that the distribution of A-WENI is statistically different from WENI (p-value = 0.04) and CC-WENI (p-value = 0.06). CC-WENI does not statistically significantly differ from WENI (p-value = 0.67).

We run the same set of analyses provided in Table 4 using the two abbreviated indices. Table 5 includes the results of those regressions; the original estimates provided in Table 4 are also include for comparison. Results are qualitatively similar between indices for all four outcomes; none of the coefficient estimates for one index are statistically different from that of another index. As with WENI, the coefficients on A-WENI and CC-WENI are statistically significantly associated with continuous BMI, MDD-W and rCSI, but are not for binomial measure of underweight. Results with coefficients on the control variables are presented in Annex Table A4.

**Table 5:** Regression results for continuous BMI, binomial BMI, MDD-W and rCSI with WENI, A-WENI and CC-WENI. Coefficient estimates are presented for OLS models and average marginal effects are presented for Probit and Poisson models.

**Variables**	**BMI** **(OLS)**			**Normal BMI = 1** **(Probit)**			**MDD-W** **(Poisson)**			**rCSI** **(OLS)**		
WENI	2.671**			0.712			0.863***			−13.70**		
	(1.039)			(0.621)			(0.237)			(5.452)		
CC-WENI		2.941**			0.767			0.794***			−15.78***	
		(1.128)			(0.661)			(0.228)			(5.750)	
A-WENI			2.487**			0.372			0.588***			−14.27**
			(0.940)			(0.640)			(0.204)			(5.514)

Observations	309	309	309	309	309	309	309	309	309	309	309	309

Ward FE	YES	YES	YES	YES	YES	YES	YES	YES	YES	YES	YES	YES
Controls	YES	YES	YES	YES	YES	YES	YES	YES	YES	YES	YES	YES
R[Pseudo-R]	0.072	0.074	0.071	[0.038]	[0.038]	[0.036]	[0.005]	[0.009]	[0.007]	0.091	0.096	0.096

Note: *** p < 0.01, ** p < 0.05, * p < 0.1.

Controls include age, age squared, marital status, an indicator on cropping, and participation in the REAP program.

Standard errors in parentheses are clustered at the manyatta level.

In sum, WENI seems to work well in Samburu, and while we replaced six indicators in WENI and added two additional indicators to reflect the local context, our results compared to results from the CC-WENI estimations in Table 5 and in Fig. 6 suggest that the overall improvement in fit associated with those efforts is limited. This should give us confidence that while researchers may still wish to tailor WENI to the local context, CC-WENI and A-WENI remain viable options as well.

***Robustness Checks***

*Level of fixed effects*

We also estimate results with manyatta (community) rather than ward fixed effects for our four outcomes of interest using the continuous WENI measure. We anticipate that shifting from ward to manyatta level fixed effects will impact the estimation results because several WENI indicators aim to capture community resources, such as the accessibility of antenatal and natal care, which will vary between manyatta. Thus, manyatta fixed effects are likely to mitigate the expression of the role of these community level characteristics on outcomes through WENI. Results with manyatta fixed effects are included in Annex Table A6. We find results are similar in magnitude to the results in Table 4, but they are not as precisely estimated.

*Including overweight and obese*

WENI was developed to focus on characteristics that are related to dietary diversity, anemia, and underweight status in South Asia (Narayanan et al. 2019). However, we can also assess whether including overweight and obese women (some of whom might in fact be pregnant) in our sample influences our findings. In Table 6, we include the 18 women who were excluded from the earlier analysis with BMI greater than 25. We find that including these women does not alter the results. With this full sample, the coefficient on WENI is significantly and positively associated with both BMI measures. Both MDD-W and rCSI are strongly associated in the expected directions with improvements in women’s empowerment. As Narayanan et al. (2019) note, the indicators included in WENI focused on undernutrition and as such are not appropriate to assess the relationship between nutritional empowerment and obesity.^10^ Therefore, the analysis itself does not allow for improved WENI to both reduce the likelihood of under and over nutrition, and so cannot speak to the role of empowerment on overnutrition.

**Table 6:** Full sample with all BMI values (Including overweight and obese individuals).

**Variables**	**BMI (OLS)**		**Normal BMI = 1 (Probit)**		**MDDS-W (Poisson)**		**rCSI (OLS)**	
WENI	7.542***	6.861***	1.460**	1.371**	0.889***	0.990***	−12.43**	−16.19***
	(1.724)	(1.716)	(0.636)	(0.641)	(0.225)	(0.227)	(4.861)	(4.790)
Observations	327	327	327	327	327	327	327	327
Ward FE	YES	YES	YES	YES	YES	YES	YES	YES
Controls	NO	YES	NO	YES	NO	YES	NO	YES
R	0.072	0.115					0.054	0.091
Pseudo-R2			0.0179	0.0474	0.0119	0.0104		

Note: *** p < 0.01, ** p < 0.05, * p < 0.1.

Controls are age, age squared, marital status, an indicator on cropping, and participation in the REAP program.

Standard errors in parentheses are clustered at the manyatta level.

## 6 Discussion and conclusion

WENI was designed to measure women’s nutritional empowerment and was previously validated in South Asia against women’s own dietary and nutritional outcomes. We find that WENI is also valid in northern Kenya, a context with substantially different livelihood opportunities, institutions, food systems, and gender dynamics than South Asia. WENI is significantly associated with both continuous measures of BMI and MDD-W. It is also associated with a household level measure of food insecurity, the rCSI. The coefficient estimates between WENI and a binary indicator of normal BMI is positive (as expected) but statistically indistinguishable from zero. The lack of statistical significance in the binary outcome could reflect that that binary outcome indicators lose precision compared to continuous measures and or due to the sample distribution in BMI around the cut-off.

Researchers interested in tracking women’s empowerment face a range of possible metrics (Santoso et al. 2019). One choice is whether to use universal or context specific empowerment measures. SWPER, for example, was intended to be universal (Ewerling et al., 2020) while others are focused on capturing more contextual aspects of empowerment, such as empowerment among women engaging in agriculture (Malapit et al. 2015), among women engaging in agricultural projects (Malapit et al. 2019), or in livestock rearing (Galiè et al. 2019). Developing an index that is applicable across contexts and outcomes is time- and resource-intensive. The process of adapting WENI to a different context has been instructive and may provide a middle ground. To validate WENI in Kenya, we maintained the original DDs while customizing several indicators that composed those domain dimensions to better reflect the local context. This allowed us to preserve the foundations of WENI, which are grounded in theory, while capturing local factors without asking culturally insensitive questions.

Nonetheless, we also find that while revising WENI to reflect the new context is useful, researchers can use a simplified version of WENI, called the CC-WENI, without significant loss of information. CC-WENI performed as well as the full WENI and requires little in the way of resource intensive contextualization. The A-WENI was meant to be an abridged version of WENI and is also context-specific, unlike CC-WENI.

Based on validation in two very different locations, the CC-WENI is intended for use when nutritional empowerment is being used as an outcome or control variable in an impact analysis; the A-WENI may be used to assess and track nutritional empowerment quickly at the community level but that the full WENI be used when objectives include improved understanding of the relationships between diet and nutrition and empowerment. In the latter case, it is critical to understand contextual factors, especially those that restrict variation in specific DDs or those that make specific DDs less relevant.

Finally, categorizing individuals is a useful way for bringing attention to specific circumstances or highlighting impacts but, when categories are based on continuous variables, they require identifying, sometimes arbitrary, thresholds. In some cases, the classifications and any conclusions drawn from them, are highly sensitive to the thresholds used, which can lead to an inaccurate interpretation of results. In this case, we found that as long as the thresholds in WENI used to classify individuals into binary classes of empowered/ unempowered maintained a reasonably large proportion of our sample in both groups, the binary indicators performed in a way that were similar to the continuous WENI index.

There are several limitations in the study. First, our sample is relatively small. Future work would benefit from a larger sample size to learn if there is indeed heterogeneity in the relationships between WENI and diet and nutrition across livelihoods and to more precisely test for differences between WENI, A-WENI, and CC-WENI. Applying WENI to locations experiencing the double burden of malnutrition (i.e., the coexistence of underweight and overweight/obese populations) is a particular priority.^11^

Second, we expect the relationship between WENI and women’s dietary and nutritional outcomes is somewhat attenuated due to the ongoing drought. While WENI was designed to measure aspects of nutritional empowerment that do not respond to short-term, seasonal shocks, the duration of this drought (four seasons at the time of data collection; see also FEWSNET 2022) appears to have adversely impacted women’s dietary and nutritional outcomes. The extremely high rates of underweight women in our sample, reaching emergency WHO thresholds, suggests that women’s BMI was uniquely poor during our survey period. We also caution that underweight women were relatively common in both the WENI South Asia and Kenya samples (Narayanan et al. 2019). BMI may be a less informative outcome indicator for other populations, such as those experiencing the double burden of malnutrition (Blackburn and Jacobs, 2014).

Finally, while the combination of theory and the empirically robust relationships between WENI and diet and nutrition are suggestive, this manuscript is unable to prove causality; it remains an open question if exogenous improvements to WENI would result in improved dietary and nutritional outcomes.

While UNICEF (2023) has projected that SDG nutritional milestones for women and adolescent girls will not be met, as the body of evidence grows, there is increasing support for the idea that women’s empowerment is important for women’s own wellbeing. Recent work by Bageant at el. (2024) indicate that different empowerment metrics seem to capture different aspects of empowerment, which indicates there is a need to continue to innovate, simplify, improve and test these metrics. We hope that an increased understanding of how to track and monitor factors associated with women’s dietary and nutritional status will help reach that goal.

The funding sources played no role in the study design; data collection, analysis or interpretation; report writing; nor the decision to submit paper for publication. The opinions expressed in this document are those of the authors.

This study received IRB approval from the University of Texas at Austin Office of Research Support and Compliance Institutional Review Board, Protocol number STUDY00000956.

Credit contributors statement

EL, SN, EB and NJ conceptualized and acquired funding for the research. All authors contributed to study design, methodology and data collection efforts. NJ and WL led the data analysis, visualization and curation, with support from EB, SN and EL, all of whom have accessed the underlying data. EL drafted the original manuscript with subsequent review and editing by SN, NJ, WL, and EB. EL led project administration.

Data sharing statement

Deidentified individual participant data, data dictionary and analysis syntax is publicly available on the Open Science Framework platform: https://osf.io/rxzm5/.

## CRediT authorship contribution statement

**Erin Lentz:** Writing – review & editing, Writing – original draft, Supervision, Project administration, Methodology, Investigation, Funding acquisition, Conceptualization. **Nathan Jensen:** Writing – review & editing, Visualization, Validation, Supervision, Software, Resources, Methodology, Funding acquisition, Formal analysis, Data curation, Conceptualization. **Watson Lepariyo:** Writing – review & editing, Writing – original draft, Visualization, Software, Formal analysis, Data curation. **Sudha Narayanan:** Writing – review & editing, Writing – original draft, Visualization, Validation, Methodology, Investigation, Funding acquisition, Conceptualization. **Elizabeth Bageant:** Writing – original draft, Supervision, Software, Methodology, Investigation, Funding acquisition, Data curation, Conceptualization.

## Declaration of competing interest

The authors declare that they have no known competing financial interests or personal relationships that could have appeared to influence the work reported in this paper.

## Data Availability

Deidentified individual participant data, data dictionary and analysis syntax is publicly available on the Open Science Framework platform: https://osf.io/rxzm5/.

## Footnotes

1 Narayanan et al. (2022) describe an extensive qualitative process to identify the ten (DD) and candidate indicators for their measurement. The fertility domain can be dropped (see Narayanan et al. 2019) and the index recalculated.

2 The Institutional Review Board at the University of Texas at Austin reviewed this study. Research clearance was provided by the Institutional Research Ethics Committee of the ILRI and the Kenyan National Commission for Science Technology and Innovation.

3 We also sought indicators that would likely have variation, although ex ante this was difficult to assess.

4 It was not appropriate for the enumerators to directly ask the respondents if they were pregnant.

5 We thank an anonymous reviewer for this point.

6 See Bageant et al. (2024) for correlation analysis between WENI and a variety of outcomes and between WENI and other empowerment measures.

7 We collected data for the nine categories of the MDD-W (2013) indicator. An alternative binary MDD-W indicator, described in FAO (2016), is generated from a 10-category dietary diversity score and equal to one if the score is greater or equal to five.

8 Polygamy is relatively common among the Samburu but it is much less so in our sample, which, as indicated above, are women identified by their communities as falling into lower wealth groups. We do not include indicators of polygamy as controls in our regression because the number reporting sharing manyattas (and therefore resources) with co-wives is extremely small (n=4).

9 Because the year of the survey coincided with a multi-season drought, we used a variable that takes a value of one if they have grown crops in the past five years, which will include some households that are not currently growing crops.

10 For example, Food Knowledge indicators focus primarily on establishing knowledge around micronutrient deficiencies or nutritive value of foods. For WENI to be applicable to settings of obesity might potentially include awareness of the nutritive value of ultra processed foods, etc.

11 We thank an anonymous reviewer for this point.
