# Supplementary material for: Adapting the Women’s empowerment in nutrition index: Lessons from Kenya
Source: World Dev. 2025 Apr;188:106887. doi: 10.1016/j.worlddev.2024.106887 (PMC11808626; doi:10.1016/j.worlddev.2024.106887)
Supplement: Supplementary Data 1 [file mmc1.docx]

# Annex

# Table A1: WENI Indicators that Replaced Original WENI Indicators

| **Domain Dimension** | **WENI Kenya Indicator** | **Revised Indicator Description** | | **Original WENI Indicator** | | **Original Indicator Description** | |
| --- | --- | --- | --- | --- | --- | --- | --- |
| **FOOD** |  |  | |  | |  | |
| **Food Knowledge** | | |  | |  | |  |
|  | FKkwashiorkor | Knows that a child's hair turning light brown is indicative of a nutritional issue of some kind | | FKCalcium | | Knowledge of foods rich in calcium | |
| **HEALTH** |  |  | |  | |  | |
| **Health Knowledge** | | |  | |  | |  |
|  | HKdiarrhea | Aware of any causes of diarrhea | | HKanemia | | Can recognize if someone has anemia | |
| **FERTILITY** |  |  | |  | |  | |
| **Fertility Knowledge** | | |  | |  | |  |
|  | TKprograms | Has knowledge about programs that help expectant mothers | | FeKmenstrpregknowledge | | Has knowledge of menstrual cycle and likelihood of pregnancy | |
|  | TKpregdiet | Has knowledge that pregnant women should eat more | | FeKpregdiet | | Is aware that pregnant women have different dietary needs | |
| **Fertility Resources** | | |  | |  | |  |
|  | TRpregless | Ate a normal amount or more in general during her last pregnancy. Or has not been pregnant | | FeRadediverse | | Had adequate and diverse diet during last pregnancy | |
| **INSTITUTIONS** | | |  | |  | |  |
|  | Ihair | Can grow long hair or not because of intrinsic motivation | | Idoveil | | Can choose to veil or not because of intrinsic motivation | |
| Note: For the original list of WENI indicators, interested readers can refer to Table 2 in Narayanan et al. (2019). We excluded "Has no dietary restrictions not of his own volition and can give these up if she wishes", an Original WENI Food Agency question because there was no variation, and we had adequate coverage in that domain dimension. | | | | | | | |

**Table A2: Descriptive statistics by ward**

| **Variables** | **Angata Nanyekie**  **(n=155)** | | **Elbarta**  **(n=92)** | | **Ndoto**  **(n=62)** | | **Total**  **(n=309)** | | |
| --- | --- | --- | --- | --- | --- | --- | --- | --- | --- |
| ***Outcome*** |  |  |  |  |  |  |  |  | |
| Mean MDD-W^1^ | 2.08 | (0.88) | 2.05 | (1.03) | 2.19 | (0.94) | 2.09 | (0.94) | |
| Mean Body Mass Index (BMI) | 18.67 | (2.25) | 18.26 | (2.57) | 18.34 | (2.23) | 18.48 | (2.35) | |
| Mean rCSI^2^ | 12.58 | (11.19) | 15.49 | (8.96) | 17.35 | (10.10) | 14.40 | (10.50) | |
| BMI Category (%) |  |  |  |  |  |  |  |  | |
| Severe thinness | 10.32 |  | 17.39 |  | 11.29 |  | 12.62 |  | |
| Moderate thinness | 13.55 |  | 21.74 |  | 20.97 |  | 17.48 |  | |
| Mild thinness | 27.74 |  | 21.74 |  | 29.03 |  | 26.21 |  | |
| Normal range | 48.39 |  | 39.13 |  | 38.71 |  | 43.69 |  | |
| ***Women Empowerment in Nutrition index (WENI)*** | |  |  |  |  |  |  |  | |
| WENI status (1=Empowered) (%) | 42.58 |  | 58.70 |  | 66.13 |  | 52.10 |  | |
| Mean WENI | 0.48 | (0.12) | 0.53 | (0.11) | 0.54 | (0.10) | 0.51 | (0.12) | |
| Mean CC-WENI | 0.47 | (0.12) | 0.54 | (0.10) | 0.54 | (0.09) | 0.50 | (0.12) | |
| Mean A-WENI | 0.46 | (0.13) | 0.51 | (0.12) | 0.50 | (0.11) | 0.48 | (0.12) | |
| ***Other variables*** |  |  |  |  |  |  |  |  | |
| REAP participation (1=Enrolled) (%) | 30.32 |  | 36.96 |  | 40.32 |  | 34.30 |  | |
| Marital status (%) |  |  |  |  |  |  |  |  | |
| Never married | 0.65 |  | 9.78 |  | 3.23 |  | 3.88 |  | |
| Married | 93.55 |  | 78.26 |  | 74.19 |  | 85.11 |  | |
| Widowed | 3.87 |  | 8.70 |  | 14.52 |  | 7.44 |  | |
| Divorced | 1.94 |  | 3.26 |  | 8.06 |  | 3.56 |  | |
| Mean Age (Completed years) | 30.28 | (7.01) | 28.42 | (5.24) | 31.02 | (6.18) | 29.88 | (6.42) | |
| ^1^Minimum Dietary Diversity score- Women (MDD-W). | | | | | | | | |  |

^2^Reduced Coping Strategy Index (rCSI).

Note: Standard deviations are in parenthesis.

**Table A3: Means of WENI Indicators**

| **Domain-Dimensions** |  | **WENI Kenya Indicator** | **Pastoral** | **Agro-pastoral** | **Total** |
| --- | --- | --- | --- | --- | --- |
| **FOOD** | | | | | |
| **Food Knowledge (2)** |  |  |  |  |  |
|  | 1 | FKkwashiorkor | 0.27 | 0.22 | 0.26 |
|  | 2 | FKiodine | 0.05 | 0.04 | 0.05 |
| **Food Resources (10)** |  |  |  |  |  |
|  | 1 | FRpaidwork | 0.24 | 0.25 | 0.24 |
|  | 2 | FRtravelforwork | 0.34 | 0.26 | 0.31 |
|  | 3 | Frland | 0.69 | 0.79 | 0.72 |
|  | 4 | FRservedlast | 0.1 | 0.16 | 0.12 |
|  | 5 | FRincomesources | 0.09 | 0.19 | 0.12 |
|  | 6 | FRselfemployment | 0.63 | 0.49 | 0.59 |
|  | 7 | FRprograms | 0.23 | 0.39 | 0.29 |
|  | 8 | FRsupportent | 0.65 | 0.39 | 0.56 |
|  | 9 | FRdiversity |  |  |  |
|  | 10 | FRlivestockcontrol | 0.9 | 0.83 | 0.87 |
| **Food Agency (5)** |  |  |  |  |  |
|  | 1 | FAdecisionpaidwork | 0.89 | 0.95 | 0.91 |
|  | 2 | FAassetconsent | 0.75 | 0.64 | 0.72 |
|  | 3 | FAagrisay | 0.99 | 0.95 | 0.97 |
|  | 4 | FAdecisionent | 0.25 | 0.3 | 0.27 |
|  | 5 | FAcashcontrol | 0.61 | 0.49 | 0.57 |
| **HEALTH** |  |  |  |  |  |
| **Health Knowledge (3)** |  |  |  |  |  |
|  | 1 | HKdiarrhea | 0.73 | 0.76 | 0.74 |
|  | 2 | HKors | 0.68 | 0.38 | 0.58 |
|  | 3 | HKmalaria | 0.39 | 0.41 | 0.39 |
| **Health Resources (5)** |  |  |  |  |  |
|  | 1 | HRworkintensity | 0.29 | 0.13 | 0.24 |
|  | 2 | HRassistwhensick | 0.72 | 0.73 | 0.72 |
|  | 3 | HRsanitation | 0 | 0.01 | 0.01 |
|  | 4 | Hrworkhours | 0 | 0.1 | 0.04 |
|  | 5 | HRworkrisk | 0.36 | 0.32 | 0.34 |
| **Health Agency (3)** |  |  |  |  |  |
|  | 1 | HAalonefortreatment | 0.97 | 0.98 | 0.97 |
|  | 2 | HApermission | 0.94 | 0.84 | 0.91 |
|  | 3 | HAdecideownhealth | 0.44 | 0.28 | 0.39 |
| **FERTILITY** |  |  |  |  |  |
| **Fertility Knowledge (2)** |  |  |  |  |  |
|  | 1 | TKprograms | 0.63 | 0.53 | 0.6 |
|  | 2 | TKpregdiet | 0.11 | 0.08 | 0.1 |
| **Fertility Resources (5)** |  |  |  |  |  |
|  | 1 | TRassistance | 0.43 | 0.22 | 0.36 |
|  | 2 | TRworkload | 0.56 | 0.43 | 0.52 |
|  | 3 | TRworkshare | 0.81 | 0.71 | 0.78 |
|  | 4 | TRqualafford | 0.44 | 0.29 | 0.39 |
|  | 5 | TRpregless | 0.24 | 0.37 | 0.28 |
| **Fertility Agency (3)** |  |  |  |  |  |
|  | 1 | TAdelivpref | 0.66 | 0.59 | 0.64 |
|  | 2 | TAchildnumber | 0.45 | 0.52 | 0.48 |
|  | 3 | TAchildspacing | 0.7 | 0.67 | 0.69 |
| **INSTITUTION (7)** |  |  |  |  |  |
|  | 1 | Igroup | 0.59 | 0.38 | 0.51 |
|  | 2 | Ihair | 0.53 | 0.78 | 0.61 |
|  | 3 | Igovinfo | 0.97 | 0.92 | 0.95 |
|  | 4 | Iviolence | 0.95 | 1 | 0.97 |
|  | 5 | Imobility | 0.68 | 0.59 | 0.65 |
|  | 6 | Icivic | 0.83 | 0.88 | 0.85 |
|  | 7 | Inetwork | 0.77 | 0.82 | 0.78 |

| **Domain-Dimensions** |  | **WENI Kenya Indicator** | **Pastoral** | **Agro-pastoral** | **Total** |
| --- | --- | --- | --- | --- | --- |
| **FOOD** | | | | | |
| **Food Knowledge (2)** |  |  |  |  |  |
|  | 1 | FKkwashiorkor | 0.27 | 0.22 | 0.26 |
|  | 2 | FKiodine | 0.05 | 0.04 | 0.05 |
| **Food Resources (10)** |  |  |  |  |  |
|  | 1 | FRpaidwork | 0.24 | 0.25 | 0.24 |
|  | 2 | FRtravelforwork | 0.34 | 0.26 | 0.31 |
|  | 3 | Frland | 0.69 | 0.79 | 0.72 |
|  | 4 | FRservedlast | 0.1 | 0.16 | 0.12 |
|  | 5 | FRincomesources | 0.09 | 0.19 | 0.12 |
|  | 6 | FRselfemployment | 0.63 | 0.49 | 0.59 |
|  | 7 | FRprograms | 0.23 | 0.39 | 0.29 |
|  | 8 | FRsupportent | 0.65 | 0.39 | 0.56 |
|  | 9 | FRdiversity | 0.19 | 0.63 | 0.34 |
|  | 10 | FRlivestockcontrol | 0.9 | 0.83 | 0.87 |
| **Food Agency (5)** |  |  |  |  |  |
|  | 1 | FAdecisionpaidwork | 0.89 | 0.95 | 0.91 |
|  | 2 | FAassetconsent | 0.75 | 0.64 | 0.72 |
|  | 3 | FAagrisay | 0.99 | 0.95 | 0.97 |
|  | 4 | FAdecisionent | 0.25 | 0.3 | 0.27 |
|  | 5 | FAcashcontrol | 0.61 | 0.49 | 0.57 |
| **HEALTH** |  |  |  |  |  |
| **Health Knowledge (3)** |  |  |  |  |  |
|  | 1 | HKdiarrhea | 0.73 | 0.76 | 0.74 |
|  | 2 | HKors | 0.68 | 0.38 | 0.58 |
|  | 3 | HKmalaria | 0.39 | 0.41 | 0.39 |
| **Health Resources (5)** |  |  |  |  |  |
|  | 1 | HRworkintensity | 0.29 | 0.13 | 0.24 |
|  | 2 | HRassistwhensick | 0.72 | 0.73 | 0.72 |
|  | 3 | HRsanitation | 0 | 0.01 | 0.01 |
|  | 4 | Hrworkhours | 0 | 0.1 | 0.04 |
|  | 5 | HRworkrisk | 0.36 | 0.32 | 0.34 |
| **Health Agency (3)** |  |  |  |  |  |
|  | 1 | HAalonefortreatment | 0.97 | 0.98 | 0.97 |
|  | 2 | HApermission | 0.94 | 0.84 | 0.91 |
|  | 3 | HAdecideownhealth | 0.44 | 0.28 | 0.39 |
| **FERTILITY** |  |  |  |  |  |
| **Fertility Knowledge (2)** |  |  |  |  |  |
|  | 1 | TKprograms | 0.63 | 0.53 | 0.6 |
|  | 2 | TKpregdiet | 0.11 | 0.08 | 0.1 |
| **Fertility Resources (5)** |  |  |  |  |  |
|  | 1 | TRassistance | 0.43 | 0.22 | 0.36 |
|  | 2 | TRworkload | 0.56 | 0.43 | 0.52 |
|  | 3 | TRworkshare | 0.81 | 0.71 | 0.78 |
|  | 4 | TRqualafford | 0.44 | 0.29 | 0.39 |
|  | 5 | TRpregless | 0.24 | 0.37 | 0.28 |
| **Fertility Agency (3)** |  |  |  |  |  |
|  | 1 | TAdelivpref | 0.66 | 0.59 | 0.64 |
|  | 2 | TAchildnumber | 0.45 | 0.52 | 0.48 |
|  | 3 | TAchildspacing | 0.7 | 0.67 | 0.69 |
| **INSTITUTION (7)** |  |  |  |  |  |
|  | 1 | Igroup | 0.59 | 0.38 | 0.51 |
|  | 2 | Ihair | 0.53 | 0.78 | 0.61 |
|  | 3 | Igovinfo | 0.97 | 0.92 | 0.95 |
|  | 4 | Iviolence | 0.95 | 1 | 0.97 |
|  | 5 | Imobility | 0.68 | 0.59 | 0.65 |
|  | 6 | Icivic | 0.83 | 0.88 | 0.85 |
|  | 7 | Inetwork | 0.77 | 0.82 | 0.78 |

Note: Some indicators have little variation in their scores (e.g., Iviolence). Because the aspects limiting empowerment vary across different populations (Kandiyoti, 1988; Kabeer 2016), we retain those indicators to enable cross-context comparisons.

**Table A4: Full regression results with all controls**

| **Variables** | **BMI (OLS)** | | | **Normal BMI =1 (Probit)** | | | | **MDDS-W (Poisson)** | | | | | | **rCSI (OLS)** | | | | | | | | |  |  |  |  |
| --- | --- | --- | --- | --- | --- | --- | --- | --- | --- | --- | --- | --- | --- | --- | --- | --- | --- | --- | --- | --- | --- | --- | --- | --- | --- | --- |
|  |  |  | |  | |  | |  | | |  | | |  | | | | |  | | | |  |  |  |  |
| WENI | 2.679** | 2.536** | | 0.775 | | 0.716 | | 0.763*** | | | 0.786*** | | | -10.14* | | | | | -11.86** | | | |  |  |  |  |
|  | (1.027) | (1.007) | | (0.607) | | (0.625) | | (0.238) | | | (0.240) | | | (5.621) | | | | | (5.612) | | | |  |  |  |  |
| REAP participation (Enrolled=1) |  | -0.421 | |  | | -0.196 | |  | | | 0.00589 | | |  | | | | | -1.867* | | | |  |  |  |  |
|  |  | (0.266) | |  | | (0.146) | |  | | | (0.0419) | | |  | | | | | (1.047) | | | |  |  |  |  |
| Age (Complete years) |  | 0.386** | |  | | 0.222** | |  | | | -0.00587 | | |  | | | | | 1.192* | | | |  |  |  |  |
|  |  | (0.174) | |  | | (0.103) | |  | | | (0.0304) | | |  | | | | | (0.609) | | | |  |  |  |  |
| Cropping (Cropping=1) |  | 1.042** | |  | | 0.491* | |  | | | 0.131** | | |  | | | | | 0.219 | | | |  |  |  |  |
|  |  | (0.494) | |  | | (0.290) | |  | | | (0.0641) | | |  | | | | | (1.951) | | | |  |  |  |  |
| Marital status (Married=1) |  | 0.251 | |  | | 0.154 | |  | | | -0.0264 | | |  | | | | | 0.947 | | | |  |  |  |  |
|  |  | (0.253) | |  | | (0.152) | |  | | | (0.0533) | | |  | | | | | (1.353) | | | |  |  |  |  |
| Age squared |  | -0.00601** | |  | | -0.0035** | |  | | | 1.39e-05 | | |  | | | | | -0.0150 | | | |  |  |  |  |
|  |  | (0.00260) | |  | | (0.00153) | |  | | | (0.00044) | | |  | | | | | (0.00959) | | | |  |  |  |  |
| Elbarta | -0.548 | 0.0856 | | -0.278 | | 0.0143 | | -0.0498 | | | 0.0230 | | | 3.431** | | | | | 4.046* | | | |  |  |  |  |
|  | (0.369) | (0.472) | | (0.169) | | (0.238) | | (0.0991) | | | (0.111) | | | (1.544) | | | | | (2.252) | | | |  |  |  |  |
| Ndoto | -0.490 | 0.114 | | -0.293 | | -0.0282 | | 0.0106 | | | 0.105 | | | 5.373*** | | | | | 5.242** | | | |  |  |  |  |
|  | (0.333) | (0.480) | | (0.180) | | (0.251) | | (0.103) | | | (0.117) | | | (1.366) | | | | | (2.177) | | | |  |  |  |  |
|  |  |  | |  | |  | |  | | |  | | |  | | | | |  | | | |  |  |  |  |
| Observations | 309 | 309 | | 309 | | 309 | | 309 | | | 309 | | | 309 | | | | | 309 | | | |  |  |  |  |
|  |  |  | |  | |  | |  | | |  | | |  | | | | |  | | | |  |  |  |  |
| Ward FE | YES | YES | | YES | | YES | | YES | | | YES | | | YES | | | | | YES | | | |  |  |  |  |
| Controls | NO | YES | | NO | | YES | | NO | | | YES | | | NO | | | | | YES | | | |  |  |  |  |
| R | 0.024 | 0.071 | |  | |  | |  | | |  | | | 0.047 | | | | | 0.080 | | | |  |  |  |  |
| Pseudo-R | |  |  | | 0.0102 | | 0.0402 | | | 0.00539 | | | 0.00816 | | | | |  | | | |  |  |  |  |  |
| Note: ***p<0.01, ** p<0.05, * p<0.1 | | | | | | | | | | | | | | |  | |  | | | |  | | |  |  |  |
| Standard errors in parenthesis clustered at the manyatta level. | | | | | | | | |  | | |  | | | |  | | | |  |  |  |  |  |  |  |

**Table A5: Testing for heterogeneity in the relationship between WENI and nutritional outcomes by livelihood group**

| **Variables** | **BMI (OLS)** | | **Normal BMI =1 (Probit)** | | **MDDS-W (Poisson)** | | **rCSI (OLS)** | |
| --- | --- | --- | --- | --- | --- | --- | --- | --- |
| WENI (Pastoral) | 2.058 | 2.248 | 0.814 | 0.830 | 1.020*** | 1.179*** | -11.48** | -15.77*** |
|  | (1.345) | (1.418) | (0.787) | (0.826) | (0.376) | (0.373) | (5.428) | (5.544) |
| WENI (Agro-pastoral) | 3.691** | 3.319** | 0.759 | 0.537 | 0.386** | 0.412** | -8.010 | -10.53 |
|  | (1.544) | (1.574) | (0.767) | (0.766) | (0.131) | (0.139) | (10.76) | (10.67) |
| Test of differences between WENI coefficients | 1.634 | 1.071 | -0.0547 | -0.294 | -0.634 | -0.768* | 3.474 | 5.243 |
|  | (2.112) | (2.142) | (1.039) | (1.055) | (0.412) | (0.404) | (11.33) | (11.64) |
|  |  |  |  |  |  |  |  |  |
| Observations | 309 | 309 | 309 | 309 | 309 | 309 | 309 | 309 |
| Ward FE | YES | YES | YES | YES | YES | YES | YES | YES |
| Controls | NO | YES | NO | YES | NO | YES | NO | YES |
| R-squared | 0.048 | 0.073 |  |  |  |  | 0.047 | 0.092 |
| Pseudo-R2 |  |  | 0.0225 | 0.0379 | 0.00816 | 0.0115 |  |  |
| Note: *** p<0.01, ** p<0.05, * p<0.1 | |  |  |  |  |  |  |  |
| Controls: age, age squared, marital status, an indicator on cropping, and participation in the REAP program | | | | | | | | |
| Standard errors in parentheses are clustered at the manyatta level. | | | | |  |  |  |  |

**Table A6: Full regression results with manyatta (community) level fixed effects**

|  | **BMI**  **(OLS)** | | **Normal BMI =1 (Probit)^#^** | | **MDD-W**  **(Poisson)** | | **rCSI**  **(OLS)** | |
| --- | --- | --- | --- | --- | --- | --- | --- | --- |
| **Variables** |  |  |  |  |  |  |  |  |
|  |  |  |  |  |  |  |  |  |
| WENI | 2.128 | 1.858 | 0.903 | 0.784 | 0.404** | 0.465** | -4.859 | -8.746 |
|  | (1.311) | (1.227) | (0.755) | (0.724) | (0.185) | (0.184) | (7.615) | (7.253) |
|  |  |  |  |  |  |  |  |  |
| Observations | 309 | 309 | 289 | 289 | 309 | 309 | 309 | 309 |
| Manyatta FE | YES | YES | YES | YES | YES | YES | YES | YES |
| Controls | NO | YES | NO | YES | NO | YES | NO | YES |
| R-squared | 0.201 | 0.239 |  |  |  |  | 0.202 | 0.235 |
| Pseudo-R2 |  |  | 0.098 | 0.131 | 0.005 | 0.046 |  |  |
| Note: *** p<0.01, ** p<0.05, * p<0.1 | | |  |  |  |  |  |  |
| Controls include age, age squared, marital status, an indicator on cropping, and participation in the REAP program. | | | | | | | | |
| Standard errors clustered at the manyatta level. | | | | | | | |  |

^#^The sample is smaller in these regressions because there are several manyattas in which there is no variation in outcomes and therefore the coefficient estimate for Normal BMI is unidentified.


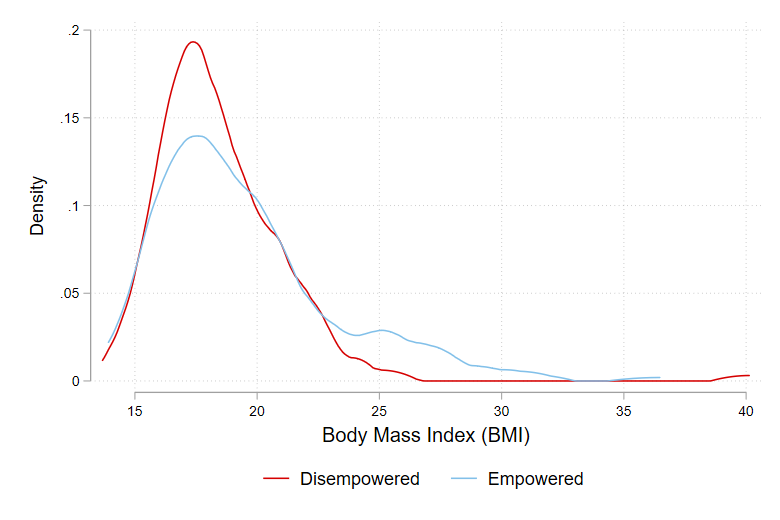


**Figure A1: Kernel Density of body mass index by empowerment status.**

**Annex 2: Survey**

**Women’s Empowerment in Nutrition Index**

Codebook

*Updated August 20, 2024*

*Contact:* [ANONYMIZED]

*This project was made possible through generous support [ANONYMIZED]*The eligibility criteria for the WENI survey are as follows.

- Age 18-49. This criterion was chosen to represent women of reproductive age, but excludes women under 18 for ethical reasons.
- Has given birth in the past 5 years. Many underlying elements of empowerment indices relate to the last or most recent pregnancy or birth. This criterion minimizes variation in time elapsed since the most recent pregnancy or birth.
- Did not report a current pregnancy at the time of interview.

| **Domain Dimension** | **WENI Kenya**  **Indicator** | **Short Label** | **Question** | **Choice set** |
| --- | --- | --- | --- | --- |
| **Health Resources** | HRsanitation | Source of drinking water | What is the main source of drinking water for your household?   Notes:   Do not include sources of water for washing, watering livestock or other non-drinking uses.   Some households will have different sources of drinking water at different times of year. Report the one that they use more than the others. | - Protected water source (piped water, borehole, water kiosk, protected well, protected spring, rainwater) - Unprotected water source (river, dam, pond, lake, stream, unprotected well, unprotected spring, water cart or truck) - Other (specify) |
| **Health Resources** | HRsanitation | Toilet | What type of toilet facility do your household members usually use? | - No facilities: Bush, field, etc (open defecation) - Unimproved toilet (open pit latrine with no slab) - Improved toilet (pit latrine with slab, flush/pour toilet) |
| **Health Resources** | HRsanitation | Cooking vent | Is there a functional vent in your cooking area that allows smoke to escape?   Notes:   A functional vent means that there is a way for the smoke to exit an indoor space so that household members do not inhale smoke.   A cooking area that is outdoors or in a shelter with no walls has a functional vent.   Observe the cooking area and probe respondent if there are inconsistencies between response and your observation. Prompt by asking whether respondent or other HH members inhale much smoke while cooking. If so, then the kitchen does not have a functional vent.   If the respondent cooks with a jiko or charcoal, and therefore does not inhale much smoke, indicate that she has a functional vent. | - No - Yes - Cooking takes place outdoors |
| **Institution** | Inetwork | Cell phone network | Is there cell phone network where you live or in any of the places that you go regularly? | - Yes - No - Don't know |
|  | FRselfemployment | Particip: staple grain farming | Did you participate in the following activities between January and December 2021?  Staple grain farming and processing of the harvest: grains that are grown primarily for food consumption (rice, maize, wheat, millet) | - Yes - No |
| **Food Resources** | FRselfemployment | Particip: horticulture or gardens | Did you participate in the following activities between January and December 2021?  Horticultural (gardens) or high value crop farming and processing of the harvest | - Yes - No |
| **Food Resources** | FRselfemployment | Particip: large livestock management | Did you participate in the following activities between January and December 2021?  Large livestock management (cattle, camels) and processing of milk and/or meat | - Yes - No |
| **Food Resources** | FRselfemployment | Particip: small livestock management | Did you participate in the following activities between January and December 2021?  Small livestock management (sheep, goats, pigs) and processing of milk and/or meat | - Yes - No |
| **Food Resources** | FRselfemployment | Particip: poultry or small animals | Did you participate in the following activities between January and December 2021?  Poultry and other small animal management (chickens, ducks, turkeys), including processing of eggs and/or meat | - Yes - No |
| **Food Resources** | FRselfemployment | Particip: fishpond culture | Did you participate in the following activities between January and December 2021?  Fishpond culture | - Yes - No |
| **Food Resources** | FRselfemployment | Particip: non-farm activities | Did you participate in the following activities between January and December 2021?  Non-farm economic activities (running a small business, self- employment, buy-and-sell) | - Yes - No |
| **Food Resources** | FRselfemployment | Particip: wage and salary employment | Did you participate in the following activities between January and December 2021?  Wage and salary employment (work that is paid for in cash or in-kind, including both agriculture and other wage work) | - Yes - No |
| **Food Resources** | FRselfemployment | Particip: other | Did you participate in the following activities between January and December 2021?  Other (specify) | - Yes - No |
| **Food Resources** | FRselfemployment | Particip: none | Did you participate in the following activities between January and December 2021?  None of these | - Yes - No |
| **Food Resources** | FRland | Land access | Do you have access to the land that you need? | - Yes - No |
| **Institution** | Igovinfo | Owns: cell phone | Do you own the [asset type] in your household? Note: Choose "Solely and jointly" if the household owns more than one [asset type] and respondent owns one or more but other household members also own this asset.  Owns: cell phone | - Yes, solely - Yes, jointly - Yes, solely and jointly - No |
| **Institution** | Imobility | Unaccompanied mpesa | Have you ever visited an Mpesa agent or a bank unaccompanied in order to conduct. An account can be used to save money, to make or receive payments, or to receive wages or financial help. | - Never - A few times - Many times - Nobody in my community can access mPesa or a bank (e.g. because of distance or connectivity) |
| **Institution** | Igroup | Group exists: ag/livestock producer | Now I'm going to ask you about groups in the community. These can be either formal or informal and customary groups. Does [group type] exist in your community, that you know of?  Group exists: ag/livestock producer | - Yes - No |
| **Institution** | Igroup | Group exists: water users' | Now I'm going to ask you about groups in the community. These can be either formal or informal and customary groups. Does [group type] exist in your community, that you know of?  Group exists: water users' | - Yes - No |
| **Institution** | Igroup | Group exists: forest users' | Now I'm going to ask you about groups in the community. These can be either formal or informal and customary groups. Does [group type] exist in your community, that you know of?  Group exists: forest users' | - Yes - No |
| **Institution** | Igroup | Group exists: credit/microfinance | Now I'm going to ask you about groups in the community. These can be either formal or informal and customary groups. Does [group type] exist in your community, that you know of?  Group exists: credit/microfinance | - Yes - No |
| **Institution** | Igroup | Group exists: mutual help/insurance | Now I'm going to ask you about groups in the community. These can be either formal or informal and customary groups. Does [group type] exist in your community, that you know of?  Group exists: mutual help/insurance | - Yes - No |
| **Institution** | Igroup | Group exists: trade/business association | Now I'm going to ask you about groups in the community. These can be either formal or informal and customary groups. Does [group type] exist in your community, that you know of?  Group exists: trade/business association | - Yes - No |
| **Institution** | Igroup | Group exists: civic | Now I'm going to ask you about groups in the community. These can be either formal or informal and customary groups. Does [group type] exist in your community, that you know of?  Group exists: civic | - Yes - No |
| **Institution** | Igroup | Group exists: religious | Now I'm going to ask you about groups in the community. These can be either formal or informal and customary groups. Does [group type] exist in your community, that you know of?  Group exists: religious | - Yes - No |
| **Institution** | Igroup | Group exists: milk/dairy marketing | Now I'm going to ask you about groups in the community. These can be either formal or informal and customary groups. Does [group type] exist in your community, that you know of?  Group exists: milk/dairy marketing | - Yes - No |
| **Institution** | Igroup | Group exists: BOMA | Now I'm going to ask you about groups in the community. These can be either formal or informal and customary groups. Does [group type] exist in your community, that you know of?  Group exists: BOMA | - Yes - No |
| **Institution** | Igroup | Group exists: youth club | Now I'm going to ask you about groups in the community. These can be either formal or informal and customary groups. Does [group type] exist in your community, that you know of?  Group exists: youth club | - Yes - No |
| **Institution** | Igroup | Group exists: political party | Now I'm going to ask you about groups in the community. These can be either formal or informal and customary groups. Does [group type] exist in your community, that you know of?  Group exists: political party | - Yes - No |
| **Institution** | Igroup | Group exists: other group | Now I'm going to ask you about groups in the community. These can be either formal or informal and customary groups. Does [group type] exist in your community, that you know of?  Group exists: Other/specify | - Yes - No |
| **Institution** | Igroup | Group exists: none in this community | Now I'm going to ask you about groups in the community. These can be either formal or informal and customary groups. Does [group type] exist in your community, that you know of?  Group exists: none in this community | - Yes - No |
| **Institution** | Igroup | Please specify other group | What is the other group? |  |
| **Institution** | Igroup | Group member | Are you a member of any of the groups you just mentioned? | - Yes - No |
| **Institution** | Igroup | Active member: ag/livestock producer | Are you an active member in the [group type]?  Active member: ag/livestock producer | - Yes - No |
| **Institution** | Igroup | Active member: water users' | Are you an active member in the [group type]?  Active member: water users' | - Yes - No |
| **Institution** | Igroup | Active member: forest users' | Are you an active member in the [group type]?  Active member: forest users' | - Yes - No |
| **Institution** | Igroup | Active member: credit/microfinance | Are you an active member in the [group type]?  Active member: credit/microfinance | - Yes - No |
| **Institution** | Igroup | Active member: mutual help/insurance | Are you an active member in the [group type]?  Active member: mutual help/insurance | - Yes - No |
| **Institution** | Igroup | Active member: trade/business association | Are you an active member in the [group type]?  Active member: trade/business association | - Yes - No |
| **Institution** | Igroup | Active member: civic | Are you an active member in the [group type]?  Active member: civic | - Yes - No |
| **Institution** | Igroup | Active member: religious | Are you an active member in the [group type]?  Active member: religious | - Yes - No |
| **Institution** | Igroup | Active member: milk/dairy marketing | Are you an active member in the [group type]?  Active member: milk/dairy marketing | - Yes - No |
| **Institution** | Igroup | Active member: BOMA | Are you an active member in the [group type]?  Active member: BOMA | - Yes - No |
| **Institution** | Igroup | Active member: youth club | Are you an active member in the [group type]?  Active member: youth club | - Yes - No |
| **Institution** | Igroup | Active member: political party | Are you an active member in the [group type]?  Active member: political party | - Yes - No |
| **Institution** | Igroup | Active member: other group | Are you an active member in the [group type]?  Active member: other group | - Yes - No |
| **Institution** | Igroup | Circumstances did join group | In general, which of the following best describe the circumstances under which you joined the group(s)? | - Of my own accord - Spouse asked me to - Social pressure - Other family member asked me to - Other circumstance (specify) |
| **Institution** | Igroup | Other circumstances: did join | What are the other circumstances? |  |
| **Institution** | Igroup | Circumstances would join | In general, which of the following best describe the circumstances under which you would join a group?    Note: If there are no groups in community and respondent indicates that she would join if a group existed, probe further to find out what would motivate her to join. | - Of my own accord - Spouse asked me to - Social pressure - Other family member asked me to - Other circumstance (specify) |
| **Institution** | Igroup | Other circumstances--would join | What are the other circumstances? |  |
| **Fertility Knowledge** | TKprograms | Program expectant mothers | Do you know about any program that helps expectant mothers?   Notes:   If respondent knows of support for pregnant women, probe them for details. If the details align with a specific program, then count the response as yes.   Main programs are:   LindaMama (NHIF) program: A public funded health scheme that will ensure that pregnant women and infants have access to quality and affordable health services.   Beyond Zero Campaign: Goal is to improve maternal and child health in Kenya, and to reduce new HIV infections among children.   CHVs do not count as a program. | Yes No |
| **Fertility Resources** | TRassistance | Government assistance last pregnancy | Did you receive assistance from the government during your last pregnancy?  Notes:   Assistance can be cash or in kind.   Examples: free delivery, free health care, transportation costs to/from delivery, baby pack, MPesa travel voucher or cash for any use. | - I received no assistance - Cash assistance only - In-kind assistance only - Both cash and in-kind assistance |
| **Fertility Resources** | TRassistance | Available: traditional birth attendant | What ante-natal, natal and post-natal health care resources are available to you where you live? Is [resource type] available?  Note: Include resources that respondent knows about and are available, even if respondent has not used them herself. | - Yes - No |
| **Fertility Resources** | TRassistance | Available: CHV | What ante-natal, natal and post-natal health care resources are available to you where you live? Is [resource type] available?  Note: Include resources that respondent knows about and are available, even if respondent has not used them herself. | - Yes - No |
| **Fertility Resources** | TRassistance | Available: community health extension worker (CHEW) | What ante-natal, natal and post-natal health care resources are available to you where you live? Is [resource type] available?  Note: Include resources that respondent knows about and are available, even if respondent has not used them herself. | - Yes - No |
| **Fertility Resources** | TRassistance | Available: health center | What ante-natal, natal and post-natal health care resources are available to you where you live? Is [resource type] available?  Note: Include resources that respondent knows about and are available, even if respondent has not used them herself. | - Yes - No |
| **Fertility Resources** | TRassistance | Available: clinic or dispensary | What ante-natal, natal and post-natal health care resources are available to you where you live? Is [resource type] available?  Note: Include resources that respondent knows about and are available, even if respondent has not used them herself. | - Yes - No |
| **Fertility Resources** | TRassistance | Available: hospital | What ante-natal, natal and post-natal health care resources are available to you where you live? Is [resource type] available?  Note: Include resources that respondent knows about and are available, even if respondent has not used them herself. | - Yes - No |
| **Fertility Resources** | TRassistance | Available: other | What ante-natal, natal and post-natal health care resources are available to you where you live? Is [resource type] available?  Note: Include resources that respondent knows about and are available, even if respondent has not used them herself. | - Yes - No |
| **Fertility Resources** | TRassistance | Available: Nothing available | What ante-natal, natal and post-natal health care resources are available to you where you live? Is [resource type] available?  Note: Include resources that respondent knows about and are available, even if respondent has not used them herself. | - Yes - No |
| **Fertility Resources** | TRassistance | Please specify the other resource | What other resource is available? |  |
| **Fertility Resources** | TRassistance | Quality: traditional birth attendant | How would you rate the quality of the ante-natal, natal and postnatal care from the [resource type]?   Note: If respondent did not use this type of care, probe to find out whether they chose to not use it because they have heard or believe that the quality is poor. | - Very good quality - Good quality - Indifferent - Poor quality - I am not able to say because I have not used this type of care and I don't know anything about it |
| **Fertility Resources** | TRassistance | Affordability: traditional birth attendant | How would you rate the affordability the ante-natal, natal and postnatal care from the [resource type]?    Note: If respondent did not use this type of care, probe to find out whether they chose to not use it because they have heard or believe that it is unaffordable. | - Very affordable or free - Somewhat affordable - Expensive, but we manage - Unaffordable - I am not able to say because I have not used this type of care and don't know anything about it |
| **Fertility Resources** | TRassistance | Quality: CHV | How would you rate the quality of the ante-natal, natal and postnatal care from the [resource type]?   Note: If respondent did not use this type of care, probe to find out whether they chose to not use it because they have heard or believe that the quality is poor. | - Very good quality - Good quality - Indifferent - Poor quality - I am not able to say because I have not used this type of care and I don't know anything about it |
| **Fertility Resources** | TRassistance | Affordability: CHV | How would you rate the affordability the ante-natal, natal and postnatal care from the [resource type]?    Note: If respondent did not use this type of care, probe to find out whether they chose to not use it because they have heard or believe that it is unaffordable. | - Very affordable or free - Somewhat affordable - Expensive, but we manage - Unaffordable - I am not able to say because I have not used this type of care and don't know anything about it |
| **Fertility Resources** | TRassistance | Quality: community health extension worker (CHEW) | How would you rate the quality of the ante-natal, natal and postnatal care from the [resource type]?   Note: If respondent did not use this type of care, probe to find out whether they chose to not use it because they have heard or believe that the quality is poor. | - Very good quality - Good quality - Indifferent - Poor quality - I am not able to say because I have not used this type of care and I don't know anything about it |
| **Fertility Resources** | TRassistance | Affordability: community health extension worker (CHEW) | How would you rate the affordability the ante-natal, natal and postnatal care from the [resource type]?    Note: If respondent did not use this type of care, probe to find out whether they chose to not use it because they have heard or believe that it is unaffordable. | - Very affordable or free - Somewhat affordable - Expensive, but we manage - Unaffordable - I am not able to say because I have not used this type of care and don't know anything about it |
| **Fertility Resources** | TRassistance | Quality: health center | How would you rate the quality of the ante-natal, natal and postnatal care from the [resource type]?   Note: If respondent did not use this type of care, probe to find out whether they chose to not use it because they have heard or believe that the quality is poor. | - Very good quality - Good quality - Indifferent - Poor quality - I am not able to say because I have not used this type of care and I don't know anything about it |
| **Fertility Resources** | TRassistance | Affordability: health center | How would you rate the affordability the ante-natal, natal and postnatal care from the [resource type]?    Note: If respondent did not use this type of care, probe to find out whether they chose to not use it because they have heard or believe that it is unaffordable. | - Very affordable or free - Somewhat affordable - Expensive, but we manage - Unaffordable - I am not able to say because I have not used this type of care and don't know anything about it |
| **Fertility Resources** | TRassistance | Quality: clinic or dispensary | How would you rate the quality of the ante-natal, natal and postnatal care from the [resource type]?   Note: If respondent did not use this type of care, probe to find out whether they chose to not use it because they have heard or believe that the quality is poor. | - Very good quality - Good quality - Indifferent - Poor quality - I am not able to say because I have not used this type of care and I don't know anything about it |
| **Fertility Resources** | TRassistance | Affordability: clinic or dispensary | How would you rate the affordability the ante-natal, natal and postnatal care from the [resource type]?    Note: If respondent did not use this type of care, probe to find out whether they chose to not use it because they have heard or believe that it is unaffordable. | - Very affordable or free - Somewhat affordable - Expensive, but we manage - Unaffordable - I am not able to say because I have not used this type of care and don't know anything about it |
| **Fertility Resources** | TRassistance | Quality: hospital | How would you rate the quality of the ante-natal, natal and postnatal care from the [resource type]?   Note: If respondent did not use this type of care, probe to find out whether they chose to not use it because they have heard or believe that the quality is poor. | - Very good quality - Good quality - Indifferent - Poor quality - I am not able to say because I have not used this type of care and I don't know anything about it |
| **Fertility Resources** | TRassistance | Affordability: hospital | How would you rate the affordability the ante-natal, natal and postnatal care from the [resource type]?    Note: If respondent did not use this type of care, probe to find out whether they chose to not use it because they have heard or believe that it is unaffordable. | - Very affordable or free - Somewhat affordable - Expensive, but we manage - Unaffordable - I am not able to say because I have not used this type of care and don't know anything about it |
| **Fertility Resources** | TRassistance | Quality: other | How would you rate the quality of the ante-natal, natal and postnatal care from the [resource type]?   Note: If respondent did not use this type of care, probe to find out whether they chose to not use it because they have heard or believe that the quality is poor. | - Very good quality - Good quality - Indifferent - Poor quality - I am not able to say because I have not used this type of care and I don't know anything about it |
| **Fertility Resources** | TRassistance | Affordability: other | How would you rate the affordability the ante-natal, natal and postnatal care from the [resource type]?    Note: If respondent did not use this type of care, probe to find out whether they chose to not use it because they have heard or believe that it is unaffordable. | - Very affordable or free - Somewhat affordable - Expensive, but we manage - Unaffordable - I am not able to say because I have not used this type of care and don't know anything about it |
| **Fertility Agency** | TAdelivpref | Place of delivery | When you delivered your last child, was the place of delivery decided on your preference? | No Yes No, but we discussed my preference |
| **Fertility Agency** | TAchildnumber | Child number decision: god | Who decides the number of children you have: [decision-maker] | Yes No |
| **Fertility Agency** | TAchildnumber | Child number decision: nature | Who decides the number of children you have: [decision-maker] | Yes No |
| **Fertility Agency** | TAchildnumber | Child number decision: husband | Who decides the number of children you have: [decision-maker] | Yes No |
| **Fertility Agency** | TAchildnumber | Child number decision: respondent | Who decides the number of children you have: [decision-maker] | Yes No |
| **Fertility Agency** | TAchildnumber | Child number decision: other family members | Who decides the number of children you have: [decision-maker] | Yes No |
| **Fertility Agency** | TAchildnumber | Child number decision: someone else | Who decides the number of children you have: [decision-maker] | Yes No |
| **Fertility Agency** | TAchildnumber | Child number decision: nobody | Who decides the number of children you have: [decision-maker] | Yes No |
| **Fertility Agency** | TAchildnumber | Child number decision: refused to answer | Who decides the number of children you have: Refused to answer | Yes No |
| **Fertility Agency** | TAchildspacing | Child spacing decision: god | Who decides on child spacing: [decision-maker] | Yes No |
| **Fertility Agency** | TAchildspacing | Child spacing decision: nature | Who decides on child spacing: [decision-maker] | Yes No |
| **Fertility Agency** | TAchildspacing | Child spacing decision: husband | Who decides on child spacing: [decision-maker] | Yes No |
| **Fertility Agency** | TAchildspacing | Child spacing decision: respondent | Who decides on child spacing: [decision-maker] | Yes No |
| **Fertility Agency** | TAchildspacing | Child spacing decision: other family members | Who decides on child spacing: [decision-maker] | Yes No |
| **Fertility Agency** | TAchildspacing | Child spacing decision: someone else | Who decides on child spacing: [decision-maker] | Yes No |
| **Fertility Agency** | TAchildspacing | Child spacing decision: nobody | Who decides on child spacing: [decision-maker] | Yes No |
| **Fertility Agency** | TAchildspacing | Child spacing decision: refused to answer | Who decides on child spacing: Refused to answer | Yes No |
| **Fertility Knowledge** | TKpregdiet | Pregnant women food | In general, do you think pregnant women need to eat more, less or the same amount of food as other adults? | More Less Same amount Other (specify) |
| **Fertility Knowledge** | TKpregdiet | Please specify other | In general, do you think pregnant women need to eat more, less or the same amount of food as other adults? Other/specify. |  |
| **Fertility Resources** | TRpregless | Last pregnancy food | Would you say you were able to get the right amount of food during your last pregnancy? | Yes No |
| **Fertility Resources** | TRpregless | Last pregnancy: less food | During your last pregnancy, did you eat less because you were pregnant? Note: If respondent ate less for some portion of pregnancy (but not all), select "I ate less in general" | I ate less in general I ate a normal amount I ate more than usual because I was pregnant Other (specify) |
| **Fertility Resources** | TRpregless | Last pregnancy: less food other | During your last pregnancy, did you eat less because you were pregnant? Other/specify. |  |
| **Health Resources** | Hrworkhours | Time spent on activity (hours) | Time use module:   Now I'd like to ask you about how you spent your time yesterday. We'll begin from yesterday midnight and continue through last night. This will be a detailed accounting. I'm interested in everything you did (i.e. resting, eating, personal care, work inside and outside the home, caring for children, cooking, shopping, socializing, etc.), even if it didn’t take you much time. I'm particularly interested in agricultural activities such as farming, gardening, and livestock management whether in the field or on the homestead. If you sometimes do more than one of these activities at the same time, please choose one as the primary thing you are doing I'm also interested in how much time you spent caring for children, especially if it happened while you did some other activity (e.g., collecting water while carrying a child or cooking while watching after a sleeping child). We will be starting at yesterday at midnight, which is in the middle of the night before yesterday started. Enumerator: Begin the interview by:   Finding out whether respondent was sleeping at midnight. If she was not sleeping, what was she doing?   Identify the time she woke in the morning, and the time she went to bed at night. These will be your reference points as you begin to understand your day. | E Work as employed F Own business work G Staple grain farming (e.g. maize, beans) H Vegetables, fruits, horticultural gardens or high value crop farming I Large livestock management (cattle, camels) J Small livestock management (sheep, goats, pigs) K Poultry and other small animals raising (chickens, ducks, turkeys) I1 Milking large livestock (cattle, camel) J1 Milking small animals (sheep, goats) Y Selling milk and other livestock products |
| **Health Resources** | HRworkintensity | Work intensity | How intense is the majority of the work that you do in a typical day at this time of year? Notes:     Keep in mind the activities that they reported in time use module and other prior questions and probe if their response is inconsistent with their activities.   Very heavy work is anything that involves lifting heavy weights, strenuous bending, pushing or pulling heavy objects, including but not limited to carrying water long distances, collecting aloe vera, cutting wood, making charcoal, constructing a house, cleaning animal sheds or work of similar intensity. | Very heavy (constructing a house, collecting aloe vera, making charcoal, cleaning animal sheds etc) Heavy (collecting firewood, carrying water >1km, watering livestock in dry season, etc) Moderate (herding, walking moderate distances, carrying water <1km, etc) Light (sitting in kiosk, caring for children, milking nearby animals, etc.) Sedentary (desk work, office work or other tasks with very little movement) |
| **Health Resources** | HRworkrisk | Work risk | In any work that you do between January 2021 and December 2021 (paid work, household enterprise, agriculture or domestic and unpaid work) would you say there is risk of physical injury, death, major health problems, sexual and/or physical abuse in one or more of the tasks that you do?     Note: This question focuses on physical risks, not psychological or economic (e.g. losing money). | No Yes, one or more of these problems exists in one or more of the tasks I do |
| **Health Resources** | HRassistwhensick | Help with chores | Do you get some help with household chores from anyone (husband, children, neighbors, other family) when you are sick? | Yes No |
| **Fertility Resources** | TRworkload | Last pregnancy: heavy work | During your last pregnancy, did you do any heavy or very heavy work at any time?   Note:   Very heavy work is anything that involved lifting heavy weights, strenuous bending, pushing or pulling heavy objects, including but not limited to carrying water long distances, collecting aloe vera, cutting wood, making charcoal, constructing a house, cleaning animal sheds or work of similar intensity.   Heavy work includes collecting firewood, carrying water &gt;1km, watering livestock in dry season, etc | No Yes, occasionally Yes, routinely |
| **Fertility Resources** | TRworkload | Last pregnancy: last month of heavy work | Until which month did you continue with these heavy or very heavy tasks? |  |
| **Fertility Resources** | TRworkshare | Last pregnancy: help with work | During your last pregnancy, to what extent did other household members or others in general share your work of all kinds? | Not at all Some Took over most/all responsibilities |
| **Food Resource** | FRpaidwork | Paid work | Have you done any paid work for someone else between January and December 2021? This does not include food-for-work programs or cash-for-work programs | Yes No |
| **Food Agency** | FAdecisionpaidwork | Why no paid work | What is the primary/main reason that you did not have any paid work between January and December 2021?  Notes:   If work was not available, select Option 1   If work was available but not suitable, select Option 1 |  |
| **Food Agency** | FAdecisionpaidwork | Why no paid work: other | What is the primary/main reason that you did not have any paid work between January and December 2021? Other/specify |  |
| **Food Agency** | FAdecisionpaidwork | Decision paid work | Was the decision to do paid work for someone else entirely your own? | Yes No Sometimes, in some cases |
| **Food Resource** | FRtravelforwork | Travel to work | In general, is it considered acceptable for women from within your community to travel more than one day's distance to do paid work outside of your community? Note: This is a general question about the community. If respondent answers for herself, probe by asking what she thinks is acceptable for other women in the community. | Yes, any woman can go if she has the opportunity No woman can do this, even if she has the opportunity Some women can do this, but it depends on the circumstances (type of work, distance, husband etc) |
| Loop through each of the following five questions for activities | | | | Crop farming  Kitchen garden  Livestock management  Poultry  Bee keeping  Non-ag/livestock other  Other |
| **Food Resources** | FRdiversity | Particip: [household work type] | Which of the following household work activities did you participate in between January and December 2021? [household work type] Notes:  If interview is taking place in the homestead, observe what activities might be taking place (e.g. kitchen garden) and confirm whether respondent participates in those activities (or maybe they are done exclusively by another household member, like a co-wife).   These activities should be work for own household, not working for other households/entities for pay. |  |
| **Food Resources** | FAdecisionent | Business ownership? [household work type] | Are [household work type] activities part of a business that specifically belongs to you or specifically belongs to someone else or belongs to the household in general?     Note: If business is part of a group (e.g. BOMA or similar) with other non -household members, then select "Business belongs to respondent and someone else jointly (e.g. BOMA business or similar)" | Business belongs to respondent Business belongs to respondent and someone else jointly (e.g. BOMA business or similar) Business belongs to someone else in household Business belongs to the household in general but not to any specific person This activity is not part of any business |
| **Food Agency** | FAdecisionent | Day-to-day decisions: [household work type] | How much say do you have in the day-to-day decisions about [household work type]? | No say Some say Major say Final/Main decision-maker |
| **Food Agency** | FAdecisionent | Major decisions: [household work type] | How much say do you have in the major decisions about [household work type]? | No say Some say Major say Final/Main decision-maker |
| **Food Resource** | FRsupportent | Received aid: [household work type] | For the [household work type] business that specifically belongs to you, did you receive aid of any kind? Aid could include a gift, grant or loan for the purpose of starting or growing a business from government, NGO, self-help group, private firms, banks, credit unions, cooperatives, a moneylender or any combination of these | Yes No |
| **Food Resource** | FRsupportent | Access to financial support | Do you believe that someone your age, gender and with your skills has access to financial support to undertake a business or economic activity? Financial resources could include gifts, grants or loans for the purpose of starting or growing a business from government, NGO, self-help group, private firms, banks, credit unions, cooperatives, a moneylender or any combination of these. | No Yes Yes, but not enough financial support / poor quality / not appropriate Yes, but hard to access financial support (distance, corruption, complexity of procedures, eligibility, etc.) Not Aware |
| **Food Agency** | FRincomesources | Independent income | Between January and December 2021 have you had any independent source of cash or in-kind income, whether earned or unearned?  Note: Consider prior responses to time use module and other work questions. If respondent mentioned activities that may earn income, probe to find out whether this income goes directly to her or not. | No (none) Cash only Cash and in-kind (mixed) In-kind only |
| **Food Resources** | FRincomesources | Independent income: Paid work | What are the sources of independent income, cash or in-kind, that you have received between January and December 2021? Has [independent income source]. Note: Because markets can be distant, if husband or other family member sells livestock on respondent's behalf and delivers cash to her, this counts as independent income. | Yes No |
| **Food Resources** | FRincomesources | Independent income: Own non-ag/livestock business | What are the sources of independent income, cash or in-kind, that you have received between January and December 2021? Has [independent income source]. Note: Because markets can be distant, if husband or other family member sells livestock on respondent's behalf and delivers cash to her, this counts as independent income. | Yes No |
| **Food Resources** | FRincomesources | Independent income: Household non-ag/livestock business | What are the sources of independent income, cash or in-kind, that you have received between January and December 2021? Has [independent income source]. Note: Because markets can be distant, if husband or other family member sells livestock on respondent's behalf and delivers cash to her, this counts as independent income. | Yes No |
| **Food Resources** | FRincomesources | Independent income: Crop cultivation | What are the sources of independent income, cash or in-kind, that you have received between January and December 2021? Has [independent income source]. Note: Because markets can be distant, if husband or other family member sells livestock on respondent's behalf and delivers cash to her, this counts as independent income. | Yes No |
| **Food Resources** | FRincomesources | Independent income: Poultry, kitchen gardens, milk, beekeeping | What are the sources of independent income, cash or in-kind, that you have received between January and December 2021? Has [independent income source]. Note: Because markets can be distant, if husband or other family member sells livestock on respondent's behalf and delivers cash to her, this counts as independent income. | Yes No |
| **Food Resources** | FRincomesources | Independent income: Livestock sales | What are the sources of independent income, cash or in-kind, that you have received between January and December 2021? Has [independent income source]. Note: Because markets can be distant, if husband or other family member sells livestock on respondent's behalf and delivers cash to her, this counts as independent income. | Yes No |
| **Food Resources** | FRincomesources | Independent income: Remittances | What are the sources of independent income, cash or in-kind, that you have received between January and December 2021? Has [independent income source]. Note: Because markets can be distant, if husband or other family member sells livestock on respondent's behalf and delivers cash to her, this counts as independent income. | Yes No |
| **Food Resources** | FRincomesources | Independent income: Parental transfers | What are the sources of independent income, cash or in-kind, that you have received between January and December 2021? Has [independent income source]. Note: Because markets can be distant, if husband or other family member sells livestock on respondent's behalf and delivers cash to her, this counts as independent income. | Yes No |
| **Food Resources** | FRincomesources | Independent income: Other family transfers | What are the sources of independent income, cash or in-kind, that you have received between January and December 2021? Has [independent income source]. Note: Because markets can be distant, if husband or other family member sells livestock on respondent's behalf and delivers cash to her, this counts as independent income. | Yes No |
| **Food Resources** | FRincomesources | Independent income: NGO or political party | What are the sources of independent income, cash or in-kind, that you have received between January and December 2021? Has [independent income source]. Note: Because markets can be distant, if husband or other family member sells livestock on respondent's behalf and delivers cash to her, this counts as independent income. | Yes No |
| **Food Resources** | FRincomesources | Independent income: Interest from deposits | What are the sources of independent income, cash or in-kind, that you have received between January and December 2021? Has [independent income source]. Note: Because markets can be distant, if husband or other family member sells livestock on respondent's behalf and delivers cash to her, this counts as independent income. | Yes No |
| **Food Resources** | FRincomesources | Independent income: Government transfer | What are the sources of independent income, cash or in-kind, that you have received between January and December 2021? Has [independent income source]. Note: Because markets can be distant, if husband or other family member sells livestock on respondent's behalf and delivers cash to her, this counts as independent income. | Yes No |
| **Food Resources** | FRincomesources | Independent income: Other (specify) | What are the sources of independent income, cash or in-kind, that you have received between January and December 2021? Has [independent income source]. Note: Because markets can be distant, if husband or other family member sells livestock on respondent's behalf and delivers cash to her, this counts as independent income. | Yes No |
| **Food Agency** | FAcashcontrol | Control income | Overall, how much control do you have over the cash and in-kind income obtained from all of these sources?  Note: If respondent has control over some sources of income but not others, select "Some control". | No control Some control Major control Full control |
| **Food Resources** | FRlivestockcontrol | Control livestock sale | How much control do you have over the herding and sale of livestock that belongs to you (solely or jointly) ? | I don't have any control over how it is herded or when to sell it I have some control over how it is herded and when to sell it I have full control over how it is herded and when to sell it I have never owned livestock (solely or jointly) |
| **Food Agency** | FAassetconsent | Control earnings use | Between January and December 2021 has it ever happened that earnings from the sale of any livestock that belongs or belonged to you (solely or jointly) has been used without your consent? | Yes No Respondent did NOT solely or jointly own livestock in past year Refused to answer |
| **Food Resources** | FRprograms | Programs: None | Has your household consistently had access to any government or NGO programs between January and December 2021? If so, which program? No program Notes:  Consistent access means used for 4 months. Do not include BOMA or other business support programs here. | Yes No |
| **Food Resources** | FRprograms | Programs: cash transfer (e.g. HSNP) | Has your household consistently had access to any government or NGO programs between January and December 2021? If so, which program? [program type] Notes:  Consistent access means used for 4 months. Do not include BOMA or other business support programs here. | Yes No |
| **Food Resources** | FRprograms | Programs: CMAM | Has your household consistently had access to any government or NGO programs between January and December 2021? If so, which program? [program type] Notes:  Consistent access means used for 4 months. Do not include BOMA or other business support programs here. | Yes No |
| **Food Resources** | FRprograms | Programs: relief food | Has your household consistently had access to any government or NGO programs between January and December 2021? If so, which program? [program type] Notes:  Consistent access means used for 4 months. Do not include BOMA or other business support programs here. | Yes No |
| **Food Resources** | FRprograms | Programs: other (specify) | Has your household consistently had access to any government or NGO programs between January and December 2021? If so, which program? [program type] Notes:  Consistent access means used for 4 months. Do not include BOMA or other business support programs here. | Yes No |
| **Health Knowledge** | HKors | ORS use | Do you know what this product is used for? [visible package of ORS on screen]  Note: Do not prompt. Correct answer is that it is used to treat diarrhea or dehydration. | Incorrect Correct |
| **Food Knowledge** | FKiodine | Goiter prevention | Which food item helps to prevent a goiter? [visible image of goiter on screen] Notes:  A goiter is a round swelling in the neck area.  Do not prompt: correct answer is iodized salt | Incorrect Correct |
| **Health Knowledge** | HKmalaria | Malaria cause | How does one get malaria? Note: Do not prompt. Correct answer is "Mosquito bite" or "infected mosquito bite" | Incorrect Correct |
| **Health Knowledge** | HKdiarrhea | Diarrhea cause | What are the ways that one can get diarrhea?  Notes:  After each response, probe by asking "Are there any other ways?" until respondent has no more responses.  Do not prompt.  Correct answers are any of the following:   Eating something contaminated   Drinking dirty/contaminated water   Eating from dirty hands or dirty utensils | Incorrect Correct |
| **Food Knowledge** | FKkwashiorkor | Hair light brown cause | If a child's hair turns to a light brown color, what does it mean?  Notes:  After each response, probe by asking ""Are there any other ways?"" until respondent has no more responses.   Do not prompt.   Correct answers are any of the following:   the child has a nutritional problem   kwashiorkor   does not eat enough protein   is eating too much of some foods (e.g. maize, ugali) and not enough of others. | Incorrect Correct |
| **Health Agency** | HAalonefortreatment | Health care choice | If you are not feeling well and need to get health care and your chores and children are cared for, and you have money to go, can your husband or anyone else in your household refuse?  Note: Unmarried or widowed women may still have someone in the household who can refuse. | No, he cannot refuse Yes, he can refuse if he has a reason I agree with Yes, he can refuse with any reason I disagree with or no reason |
| **Health Agency** | HApermission | Clinic alone | Can you go alone to the health center (CHV, clinic, hospital)?  Note: "Going alone" in this question means going without someone supervising or controlling you (not bringing a friend or family member for support). | Yes No |
| **Health Agency** | HAdecideownhealth | Health care decision | Who usually makes decisions for your health care? For example, decisions about whether to seek health care, where to go for care, how to go there, costs related to care, etc. | Respondent makes decision alone Respondent and someone else (or multiple people) make decision together Someone else (or multiple people) make decision without respondent Decision not applicable |
| **Food Agency** | wn_fafooddistribution  _newvar | Food in hh decision | How much say do you have in how cooked food is served within your household? | No say Some say Major say Final/Main decision-maker |
| **Food Agency** | FRservedlast | Served last | How often would you say you are served last, including when you serve yourself? | Always Usually Sometimes Rarely Never |
| **Food Agency** | FAagrisay | Sell milk | In general, how much say do you have in whether to sell milk produced by your household? This includes the decision to not sell milk.  Notes:  If household does not produce milk at any time between January and December 2021, select N/A.   If household does not have a place to sell milk at all between January and December 2021, select N/A. | No say Some say Major say Final/Main decision-maker Not applicable (e.g. household does not produce milk or household does not have a place to sell milk) |
| **Institution** | Ihair | Long hair why | What is the primary reason that motivated you to grow your hair long? Note: Probe respondent for all reasons and select the primary one. | Because I feel like it Because I believe it is the right thing to do Because this is what is commonly done in my community or family Because I don't want to offend the community or family Because if I don't, I may face trouble, restrictions, abuse, etc. Other reason (specify) |
| **Institution** | Ihair | Long hair why other | What was the other reason? |  |
| **Institution** | Imobility | Visit parents | If you wish to visit your parents and your chores and children are cared for, and you have money to go, can your husband or anyone else in your household refuse?  Note: Unmarried or widowed women may still have someone in the household who can refuse. | No, he cannot refuse Yes, he can refuse if he has a reason I agree with Yes, he can refuse with any reason I disagree with or no reason |
| **Institution** | Iviolence | DV help: I have no one | If your husband or another family member mistreats you in one of the above ways and it is too much or goes beyond what is acceptable who would you go to for help? [person type] | Yes No |
| **Institution** | Iviolence | DV help: Refused to answer | If your husband or another family member mistreats you in one of the above ways and it is too much or goes beyond what is acceptable who would you go to for help? Refused to answer. | Yes No |
| **Institution** | Iviolence | Other person for help | If your husband or another family member mistreats you in one of the above ways and it is too much or goes beyond what is acceptable who would you go to for help? Other/specify. | Yes No |
| **Institution** | Icivic | Past 5 years: None of these | Which of the following have you participated in at least once during the past five years? No civic activity. | Yes No |
| **Institution** | Icivic | Info on gov't initiatives: local language radio | Where can you get information about government schemes or initiatives? This can include cash or in-kind benefit programs, business support programs or any other kind of assistance. [information source] | Yes No |
| **Institution** | Icivic | Info on gov't initiatives: national radio | Where can you get information about government schemes or initiatives? This can include cash or in-kind benefit programs, business support programs or any other kind of assistance. [information source] | Yes No |
| **Institution** | Icivic | Info on gov't initiatives: local elder/chief | Where can you get information about government schemes or initiatives? This can include cash or in-kind benefit programs, business support programs or any other kind of assistance. [information source] | Yes No |
| **Institution** | Icivic | Info on gov't initiatives: NGO | Where can you get information about government schemes or initiatives? This can include cash or in-kind benefit programs, business support programs or any other kind of assistance. [information source] | Yes No |
| **Institution** | Icivic | Info on gov't initiatives: billboard or flyer | Where can you get information about government schemes or initiatives? This can include cash or in-kind benefit programs, business support programs or any other kind of assistance. [information source] | Yes No |
| **Institution** | Icivic | Info on gov't initiatives: mobile phone | Where can you get information about government schemes or initiatives? This can include cash or in-kind benefit programs, business support programs or any other kind of assistance. [information source] | Yes No |
| **Institution** | Icivic | Info on gov't initiatives: husband or other household members | Where can you get information about government schemes or initiatives? This can include cash or in-kind benefit programs, business support programs or any other kind of assistance. [information source] | Yes No |
| **Institution** | Icivic | Info on gov't initiatives: another source (specify) | Where can you get information about government schemes or initiatives? This can include cash or in-kind benefit programs, business support programs or any other kind of assistance. [information source] | Yes No |
| **Institution** | Igovinfo | Info on gov't initiatives: no source of information | Where can you get information about government schemes or initiatives? This can include cash or in-kind benefit programs, business support programs or any other kind of assistance. [information source] | Yes No |
| **Institution** | Igovinfo | Please specify the other source | Where can you get information about government schemes or initiatives? This can include cash or in-kind benefit programs, business support programs or any other kind of assistance. Other/specify. |  |
